# Which Symptoms, Complaints and Complications of the Gastrointestinal Tract Occur in Patients With Eating Disorders? A Systematic Review and Quantitative Analysis

**DOI:** 10.3389/fpsyt.2020.00195

**Published:** 2020-04-20

**Authors:** Caroline Riedlinger, Greta Schmidt, Alisa Weiland, Andreas Stengel, Katrin Elisabeth Giel, Stephan Zipfel, Paul Enck, Isabelle Mack

**Affiliations:** ^1^Department of Psychosomatic Medicine and Psychotherapy, University Hospital Tübingen, Tübingen, Germany; ^2^Competence Center for Eating Disorders (KOMET), Tübingen, Germany

**Keywords:** eating disorder (ED), binge eating disorder (BED), systematic review, bulimia nervosa, bulimia nervosa, gastrointestinal symptom, gastrointestinal complaint, gastrointestinal complication

## Abstract

**Background:**

Eating disorders (ED) such as anorexia nervosa (AN), bulimia nervosa (BN), and binge eating disorder (BED) are often accompanied by a variety of psychological and physical comorbidities. Gastrointestinal (GI) symptoms are a classical feature in most patients with ED. The heterogeneity of studies on this topic is high, making it difficult to have a clear overview. The aim of this systematic review is therefore to provide an overview of subjectively and objectively measured differences and changes in the GI tract in patients with EDs, along with the occurrence of GI complications.

**Methods:**

A systematic literature search was conducted in PubMed, Web of Science, and Google Scholar to find all relevant studies examining GI problems in AN, BN, and BED. Quantitative analyses were performed for objective GI physiology measures where applicable.

**Results:**

The review differentiated between ED types and also between studies that report GI outcomes of ED in (i) human studies with an ED diagnosis excluding case reports that provide an overview of GI problems in ED and (ii) case reports with an ED diagnosis describing rare GI complications in ED. GI symptoms and impaired gastric transit times were frequent features of EDs with specific differences found for the ED types. During the time course of treatment, GI symptoms changed and/or improved but not completely. GI complications extended the range of GI problems observed, including a variety of serious complications such as gastric dilatation.

**Conclusions:**

Problems of the GI tract are frequent in patients with ED and it is likely that they complicate therapy, especially in patients with AN.

**Systematic Review Registration:**

PROSPERO registration number: CRD42019100585.

## Introduction

Eating disorders (ED) include anorexia nervosa (AN), bulimia nervosa (BN), binge eating disorder (BED) and, Other Specified Feeding and Eating Disorders (OSFED) according to the Diagnostic and Statistical Manual of Mental Disorders 5 (DSM-5) criteria (1) as well as the International Classification of Diseases 11 (ICD-11) (2). EDs can be associated with being underweight or overweight and obese but also with normal weight.

AN [Body Mass Index (BMI) < 18.5 kg/m^2^] is the best known ED and, together with substance abuse disorders, has the highest mortality rate of all mental illnesses (3–5). The urge to be “thin” and the fear of becoming “fat” are the driving forces for restrictive eating behavior (restrictive type) and often for other behaviors, e.g. excessive physical activity, which sometimes leads to extreme and life-threatening weight loss. Some patients regularly have binge eating episodes which are counteracted by self-induced vomiting, laxative and diuretic abuse (bulimic or binge/purging type) (1, 2). The prevalence of AN ranges between 0.9–4% in women and is about 0.3% in men (6–8).

In BN, patients are usually of normal weight and suffer from regular binge eating episodes with loss of control. This means that very large amounts of food (3000 to 5000 kcal) are consumed without interruption in a rather short period of time. Self-induced vomiting and the misuse of laxatives and other drugs serve as compensation (2). The prevalence of BN has recently been summarized as <1-2% (6).

Similarly, patients with BED have regular binge eating episodes at least once a week over a period of 3 months with loss of control. However, there is generally no compensatory behavior and many of these patients are overweight (BMI > 25 kg/m^2^) or obese (BMI > 30kg/m^2^) (1, 2). The prevalence of BED is <1-4% (5).

Due to the disturbed eating and food intake behavior, EDs are often associated with being under- or overweight and are accompanied by a variety of comorbidities, especially gastrointestinal (GI) tract problems (9, 10). Functional GI disorders are therefore a standard feature in most patients with EDs. EDs are associated with altered GI physiology and microbiota (11, 12). These GI problems can complicate the treatment of ED and the GI symptoms shift during the course of recovery. Some remain, others disappear, and others develop (11, 13–15). An important underlying mechanism for these observations is the microbiota-gut-brain-axis which allows bidirectional communication between the central nervous system and the gut (16, 17). Janssen (15) therefore postulated that GI physiology disturbed by an ED, in combination with other mental illnesses, could lead to the maintenance or intensification of symptoms (15).

Studies reporting GI symptoms, complaints and complications in patients with ED are extremely heterogeneous in their aims, design, participants, measurements, treatments, and outcomes (18) making it difficult to maintain a clear overview of the topic. A systematic review of the literature across the range of EDs and the GI tract is currently lacking in the literature. The objective of this review was to provide a systematic overview over the topic, applying broad inclusion criteria in order to capture the whole picture of GI problems and complications in patients with AN, BN, and BED.

## Methods

To conduct and report the systematic review, we applied the Preferred Reporting Items for Systematic Reviews and Meta-Analyses (PRISMA) statement criteria (19, 20). The review protocol is registered in the International Prospective Register of Systematic Reviews (PROSPERO; CRD42019100585).

### Literature Information Sources and Search Strategy

The review process was conducted and reported on the basis of the PRISMA statement (19, 20). PubMed and Web of Science databases were searched for literature on 3 March 2018 and 6 March 2018, respectively, and on 1 November 2019 for an update. We built two similar search terms for the two databases to find all articles related to EDs and GI problems.

The following search terms were used for 1) PubMed: ((((((((anorexia nervosa[MeSH Terms]) OR bulimia nervosa[MeSH Terms]) OR binge-eating disorder[MeSH Terms]) OR anorexia nervosa) OR bulimia nervosa) OR binge-eating)) AND ((((gastrointestinal tract[MeSH Terms]) OR gastrointestinal disease[MeSH Terms]) OR gastrointestinal) OR intestinal)) AND (((((comorbidity[MeSH Terms]) OR ((signs and symptoms[MeSH Terms]))) OR comorbidity) OR symptoms) OR complaints) and 2) Web of Science: (TS=(anorexia nervosa) OR TS=(bulimia nervosa) OR TS=(binge eating)) AND (TS=(gastrointestinal tract) OR TS=(gastrointestinal diseases) OR TS=(gastrointestin*) OR TS=(intestin*)) AND (TS=(comorbidit*) OR TS=(symptom*) OR TS=(complaint*).

Additionally, we conducted a grey literature search by analyzing the first 200 search results on Google Scholar using the search terms ‘eating disorders’ and ‘gastrointestinal symptoms’ on 8 March 2018.

### Eligibility Criteria

Eligibility criteria were based on the five PICOS dimensions, i.e., participants (P), interventions (I), comparators (C), outcome (O), and study design (S) (21, 22). We modified the common PICOS scheme by inserting the item ‘investigations’ instead of ‘interventions’ for correctness.

*Participants*: Studies were included if they were conducted in patients with AN, BN, or BED, or if the authors reported a secured ED diagnosis with common features of the aforementioned EDs but not necessarily exactly stating which ED. Studies exclusively conducted in patients with OSFED were excluded. To meet the inclusion criteria, at least one participant above 16 years of age per study was necessary. Thus, some articles may also include children younger than 16 years of age. No restrictions were made regarding ethnicity, sex, or health status.

*Investigations*: Studies diagnosing or assessing at least one symptom, problem, disturbance, or complication of the GI tract, which we defined as teeth, mouth, salivary glands, pharynx, esophagus, stomach, gut with small intestine (duodenum, jejunum, ileum), and large intestine (caecum, colon, sigmoid, rectum), and the anus. Studies were excluded if they dealt with symptoms presumably provoked by any other abdominal organ (e.g. liver, pancreas, gallbladder), misdiagnosis of independent GI diseases, or exclusively with GI hormones.

*Comparators*: A control group of any health status was not necessary in order to meet the inclusion criteria but was allowed.

*Outcome measures*: All measures which are available to assess subjective and/or objective GI symptoms, problems, disturbances or complications.

*Study design*: In order to provide a broad overview of the topic, studies with original data were not excluded because of their study design or methodology. Thus, randomized and non-randomized, quantitative and qualitative studies with and without comparison groups, pre-post designs (with follow-up) and observational studies with any sample size (including case reports) were included.

In the screening process, articles were not considered for further evaluation if they were not peer-reviewed such as books, letters, meeting abstracts, editorials, guidelines, or if they did not report primary data (e.g. reviews). Articles meeting all criteria and written either in English, German, Spanish, or French were included. Altogether, no restrictions were imposed regarding formal study characteristics such as length of follow-up, study setting, different interventions, and outcome measures, as long as outcomes regarding the GI tract were reported to be associated with ED.

*Subgroup*: The possibility of performing meta-analyses on certain GI outcomes was considered but was not feasible because only four randomized controlled trials were found, in which different GI outcomes were assessed. Instead, quantitative subgroup analyses of patients with EDs versus healthy participants were performed for the following subgroups of different GI outcomes: gastric half-emptying time, serum amylase levels, and salivary flow rate. For these analyses, studies were included if the required data were reported in the text, in tables, or in figures for patients and for healthy controls. For gastric half-emptying time, studies assessing this value for liquids and solid foods by scintigraphy were considered. Studies reporting other variables for gastric emptying time which could not be converted into gastric half-emptying time or applying other techniques for gastric emptying measurement were excluded. For a subgroup analysis of serum amylase levels, studies reporting data of total serum amylase levels were included. Total serum amylase consists of salivary and pancreatic serum amylase and was reported more frequently than salivary serum amylase. Salivary and total serum amylase can be analyzed interchangeably considering our included studies, as no elevated pancreatic serum amylase levels or pancreatic damage was reported. Therefore, each included study concluded that salivary serum amylase was elevated and that elevated total serum amylase levels could not be of pancreatic origin. Subgroup analyses for salivary flow rate were conducted for studies assessing the parotid glands during resting conditions and stimulation by citric acid. Studies were excluded if it was not reported which salivary gland was investigated.

### Study Selection, Data Collection, and Organization

For study selection and data collection, we used a modified PICOS scheme (21, 22). After initial literature search the duplicates were removed before titles and abstracts were screened independently by the first two authors (CR and GS) for potential inclusion and discussed in case of conflict. The first authors agreed in 96.2% (1026) of studies. A third reviewer (IM) was consulted for the remaining studies. For eligibility 364 full-text articles were evaluated. To provide a structured overview, we distinguished between subjective and objective measures and outcomes, respectively.

Due to the large variety of study types, the articles were categorized into two groups:

1. Human studies with ED diagnosis, excluding case reports: This group included articles with all types of study designs except for case reports where the diagnosis of ED was specified according to the authors, and participants were assessed for GI disturbances. Group 1 was further divided into the specific EDs: AN, BN, and BED.
2. Case reports with ED diagnosis: These articles were handled separately because they showed a very different profile of GI outcomes compared with the other studies categorized into group 1. These reports point out GI complications in EDs in particular.

### Data Items and Statistics

The following information was extracted from each included article for groups 1 and 2: year of publication, ED categorization, study type, follow-up/study length, sample characterization including sample size, sex, age, and BMI, diagnostic method, subjective and objective GI problems, and intervention. In addition to these items, ED diagnostic criteria for group 1 and lethal outcomes for group 2 are reported. To provide an overview across studies, mean (weighted for the number of participants per study) and median [interquartile range] were calculated for sample size, age, BMI, and percentage of male participants for all EDs, and also separately for each ED in group 1.

For quantitative subgroup analyses, mean values ± standard deviation for gastric half-emptying time (in min), serum amylase levels (in U/L) or salivary flow rate (in ml/min) as well as sample sizes of ED participants and healthy controls were extracted from all studies eligible for subgroup analysis. To provide a summary of the data across the studies, the values of interest were multiplied by the number of patients or healthy controls in the respective study and divided by the total number of participants or healthy controls, respectively. Finally, the total mean was calculated as a weighted sum of values from the individual studies.

### Risk of Bias

For each study in group 1, a risk of bias assessment was performed using the Office of Health Assessment and Translation (OHAT) Risk of Bias Rating Tool for Human and Animal Studies checklist (23). Depending on the study type, different (applicable) items are evaluated as described in the OHAT checklist. The items include questions asking for adequate randomization, allocation to appropriate comparison groups and the accounting for confounding and modifying variables, among others. Each item is rated with one of four options: definitely low (++), probably low (+), probably high (-), or definitely high risk of bias (- -). The items were not numerically summarized into a final score for single studies in accordance with the PRISMA statement (19, 20). However, an overview for the risk of bias across the studies is reported in the results section. Risk of bias was not assessed for group 2 because we considered that the risk of bias is overall high for case reports. Articles in group 1 with a high risk of bias were not excluded since we aimed at making the overview as broad as possible.

## Results

### Study Selection and Categorization

**Figure 1:**
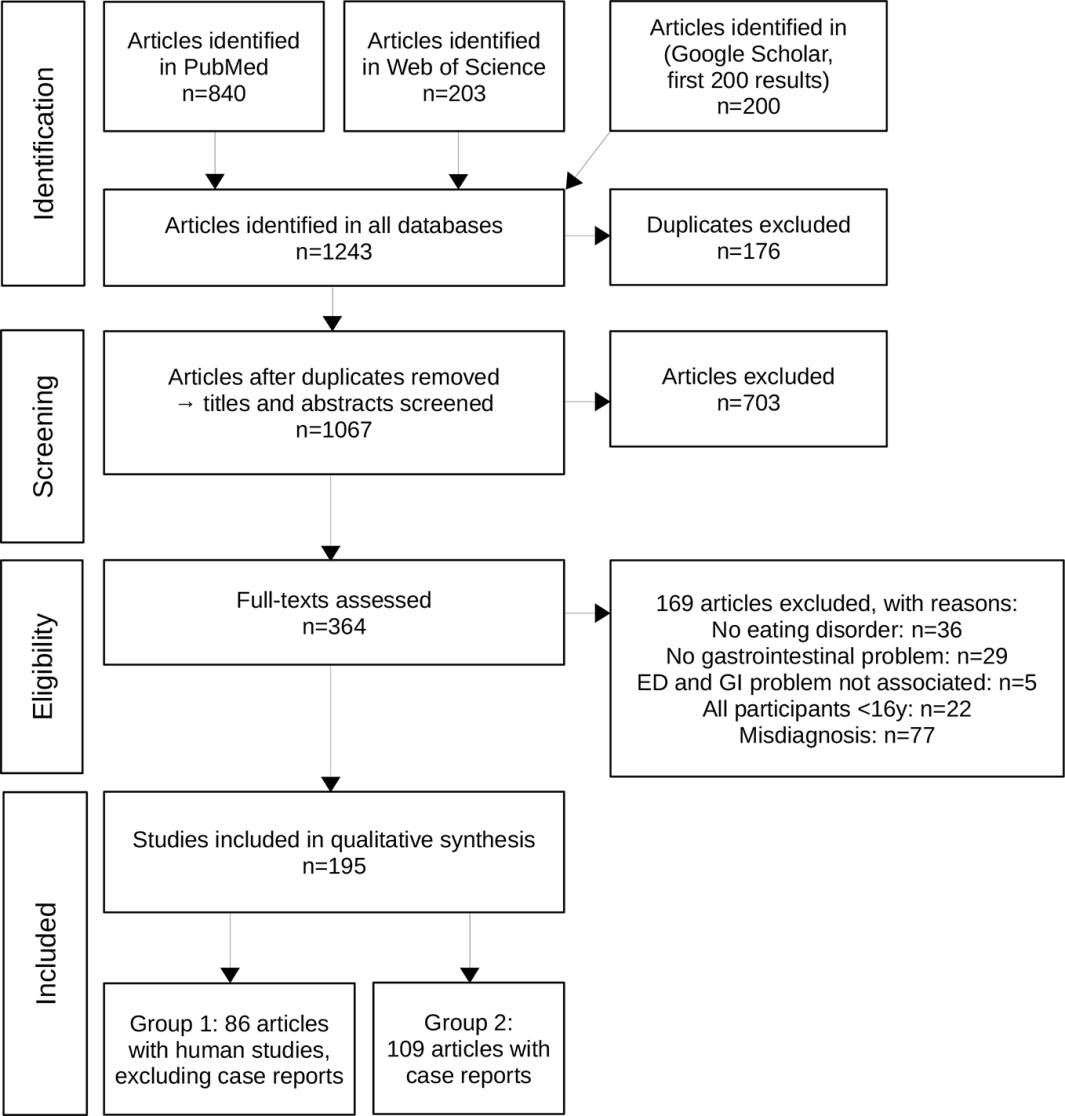
The PRISMA flowchart shows the process of study inclusion in detail with number of studies (n) included or excluded at every step of the process.

The detailed study selection process of our systematic literature search is shown in **Figure 1**. A total of 195 articles met the inclusion criteria. The key information from the single studies is presented in the **Supplementary Material Tables 1** and **2** due to the large amount of data. Eighty-six articles were categorized into group 1 (9, 14, 24–106), which comprises human studies with ED diagnosis (without case reports). Group 1 was further divided into the different EDs namely AN, BN, and BED as depicted in **Supplementary Material Table 1**. Group 2 consists of 109 case reports with a clear ED diagnosis (107–215) as shown with separate subheadings for the different EDs in **Supplementary Material Table 2**. This group in particular comprises studies describing rare complications of ED.

### Summary of Study Characteristics

#### Group 1—Human Studies With ED Diagnosis, Excluding Case Reports

The articles in group 1 were published between 1967 and 2019 (AN: 1967-2019, BN: 1968-2014, BED: 1992-2017). Out of 86 studies, AN of any subtype was assessed in 52, BN in 48, and BED in 9 studies. Thirty-three were mixed studies that assessed at least two different EDs (or AN subtypes). **Table 1** summarizes the study characteristics of group 1 in terms of sample size and participants (sex, age, BMI) across all studies in group 1 and for each ED separately. Detailed information on study design among articles in group 1 are also shown in **Supplementary Material Table 1**. Sixty-seven out of 83 studies in group 1 included a non-ED control group. Only five studies used a kind of randomization. Pre-post designs were found in 30 of the studies (34.9%) of which 28 conducted an intervention. Study length was only reported for 23 of the pre-post studies of which four studies only reported the length range. Of the remaining 19 studies the median study length was 84 days [IQR=42-154].

**Table 1:** Group 1: Summary of study characteristics.

Sample size	Mean	Median	IQR	Minimum	Maximum
					
All EDs (n = 86, r = 86)	55.5	24.5	[13.3 - 46.5]	3	850
AN (n = 53, r = 52)	37.3	18.0	[10.5 - 30.8]	3	293
BN (n = 46, r = 45)	21.7	16.5	[10.0 - 33.0]	2	66
BED (n = 9, r = 9)	171.8	56.0	[11.0 - 111.0]	6	850
Controls (n = 67, r = 66)	482.7	25.5	[14.0 - 56.5]	5	14647
**Sex (% males)**					
All EDs (n = 86, r = 77)	5.5	0.0	[0.0 - 6.3]	0.0	50
AN (n = 53, r = 44)	3.3	0.0	[0.0 - 8.2]	0.0	28.6
BN (n = 46, r = 36)	1.9	0.0	[0.0 - 0.0]	0.0	16.7
BED (n = 9, r = 6)	5.7	2.3	[0.0 - 8.8]	0.0	53.3
Controls (n = 67, r = 55)	24.0	0.0	[0.0 – 16.7]	0.0	100
**Age (years)**					
All EDs (n = 86, r = 70)	23.5	24.0	[22.0 - 26.6]	15.5	45.1
AN (n = 53, r = 38)	24.6	23.4	[19.8 - 26.3]	15.0	32.0
BN (n = 46, r = 29)	25.1	24.4	[23.1 - 25.8]	16.5	32.0
BED (n = 9, r = 3)	44.3	45.1	[37.1 - 45.1]	29.0	45.1
Controls (n = 67, r = 46)	21.9	26.0	[22.9 - 28.2]	11.7	41.4
**BMI (kg/m²)**					
AN (n = 53, r = 21)	14.5	15.1	[14.0 - 16.0]	12.0	19.2
BN (n = 46, r = 17)	22.1	22.3	[21.7 - 22.5]	15.1	22.9
BED (n = 9, r = 3)	28.0	31.1	[29.0 - 33.9]	26.9	36.6
Controls (n = 67, r = 26)	25.3	22.1	[21.4 - 22.7]	14.6	35.5

n = total amount of studies in this group, r = amount of studies reporting the variable.

#### Group 2—Case Reports With ED Diagnosis

The articles in group 2 were published between 1968 and 2019. Group 2 consists of 109 case report studies, of which 94 reported only one patient case, while 15 reported more than one case. Separated by ED types, AN restrictive subtype was reported in 16, AN binge/purge subtype in 44, BN in 25, and BED in 0 studies. The AN subtype was not clearly presented in 14 studies, and another 11 studies did not define the difference between AN with binge/purge subtype and BN. In total, 142 patient cases (130 [91.5%] females, 12 [8.5%] males) are reported in group 2. The mean age of all patients reported in group 2 was 25.8 years (median=24 [19-30]; age was not reported in two articles). The BMI was only reported in 68 out of 142 cases. Therefore, weight status was also reported in **Supplementary Material Table 2** as the lowest noted body weight (BW) or % of ideal body weight (% IBW) if BMI data were not available. Individual weight status results are presented in **Supplementary Material Table 2** for each study separately.

### Summary of Study Outcomes

GI outcome measurements were extremely heterogeneous among all study groups. Subjective outcomes were assessed by self-constructed questionnaires as well as a great variety of validated questionnaires. Objective measurements ranged from physical examination to a variety of technical methods such as serum and saliva tests, radiographic and endoscopic methods, as well as the measurement of specific GI transit times, manometric measurements, and intraoperative diagnostics.

#### Overview of Study Outcomes at a Qualitative Level for Group 1—Human Studies With ED Diagnosis, Excluding Case Reports

**Figure 2:**
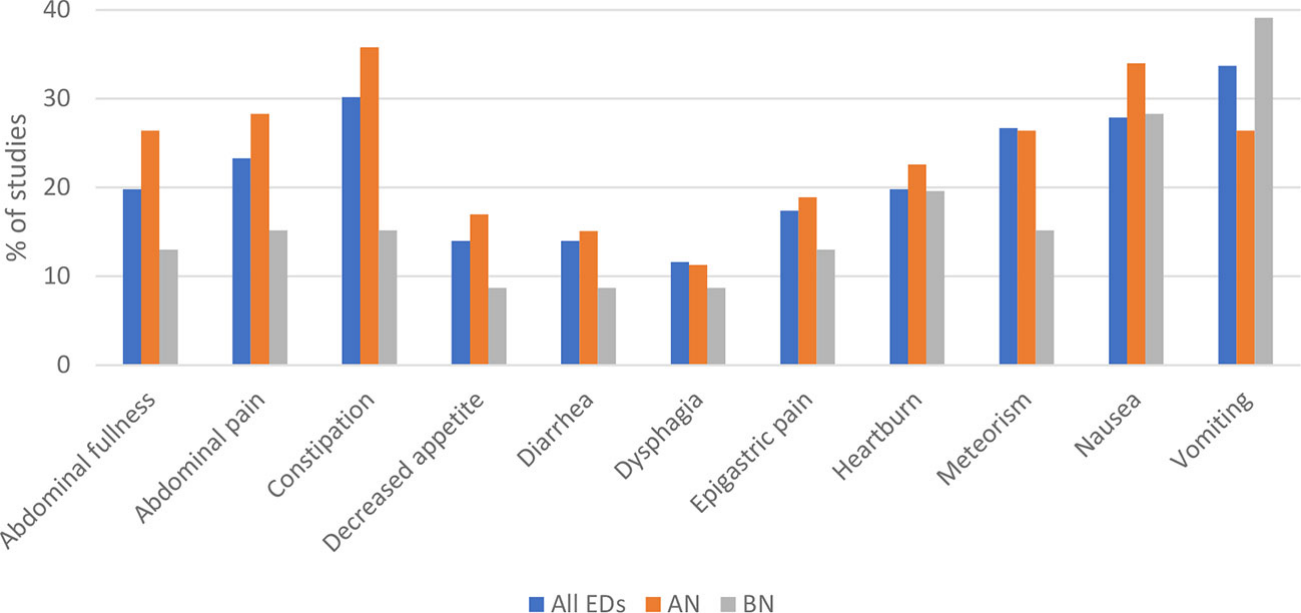
Subjectively reported gastrointestinal (GI) outcomes of group 1 studies that were reported most frequently are presented for all eating disorders (ED), Anorexia nervosa (AN), and Bulimia nervosa (BN). GI symptoms presented for ED are not the calculated sums of separate EDs shown in this figure. It is the summary of studies shown in table 1 of the supplements which assessed more than one ED. The data of the figure are presented below as sample size/% of studies reporting the symptom. Most frequently reported subjective outcomes among all EDs were vomiting (29/33.7%), constipation (26/30.2%), nausea (24/27.9%), meteorism (23/26.7%), abdominal pain (20/23.3%), heartburn (17/19.8%), abdominal fullness (17/19.8%), epigastric pain (15/17.4%), diarrhea (12/14.0%), decreased appetite (12/14.0%), and dysphagia (10/11.6%). For each ED separately, most frequent GI outcomes were in AN: constipation (19/35.8%), nausea (18/34.0%), abdominal pain (15/28.3%), abdominal fullness (14/26.4%), meteorism (14/26.4%), vomiting (14/26.4%), heartburn (12/22.6%), epigastric pain (10/18.9%), decreased appetite (9/17.0%), diarrhea (8/15.1%), and dysphagia (6/11.3%); and in BN: vomiting (18/39.1%), nausea (13/28.3%), heartburn (9/19.6%), abdominal pain (7/15.2%), constipation (7/15.2%), meteorism (7/15.2%), abdominal fullness (6/13.0%), epigastric pain (6/13.0%), decreased appetite (4/8.7%), diarrhea (4/8.7%), and dysphagia (4/8.7%).

Outcomes and measurements of group 1 are presented individually in **Supplementary Material** **Table 1** and the outcomes are also summarized in **Figure 2** and **Table 2**.

**Table 2:** Group 1: Objective GI problems reported by human studies with ED diagnosis, excluding case reports.

	ED		AN		BN	
	n	%	n	%	n	%
Caries	2	2.3	0	0	2	4.3
Delayed gastric emptying	18	20.3	13	24.5	7	15.2
Dental erosion	4	4.7	1	1.9	4	8.7
Esophagitis	4	4.7	2	3.8	3	6.5
Gastric electrical dysrhythmia	3	3.5	3	5.7	2	4.3
Gastroesophageal reflux	4	4.7	2	3.8	2	4.3
Hyperamylasemia	4	4.7	3	5.7	4	8.7
Low salivary flow rate	5	5.8	0	0	5	10.9
Salivary gland hypertrophy	2	2.3	1	1.9	2	4.3

Objective GI outcomes which were reported most frequently are presented for all eating disorders together (ED), Anorexia nervosa (AN) and Bulimia nervosa (BN). The number (n) and percentage (%) of studies reporting the specific GI outcome are shown. GI symptoms presented for ED are not the calculated sums of separate EDs shown in this figure. It is the summary of studies shown in **Supplementary Material Table 1** which assessed more than one ED.

*Subjective GI outcomes* were assessed in 47 studies using either self-constructed (n=34) or validated questionnaires (n=20), or both. The outcomes varied from abdominal pain and nausea to constipation and diarrhea. Patterns of GI symptoms differed between the different EDs. The data are depicted in **Figure 2**, except for BED, due to the small number of studies available. The most commonly reported GI symptom in AN was constipation (19/35.8%) and vomiting in BN (18/39.1%). GI problems reported in BED were abdominal fullness (4/44.4%), epigastric pain (4/44.4%), and nausea (4/44.4%).

*Objective GI outcomes* were measured in 57 studies with many different clinical methods, among which gastric emptying was reported most frequently (in 19 studies). Other frequently reported measurements were dental/oral examination (11/12.8%), salivary flow rate (5/5.8%), and serum amylase (5/5.8%). Objective GI outcomes varied from different delayed bowel transit times and esophageal reflux to increased stomach capacity. An overview of the most common outcomes for ED in general, AN, and BN is presented in **Table 2**. In BED, specific objective GI symptoms were not reported in more than one study and the individual outcomes can be seen in **Table 1** of the Supplementary Material. As depicted in **Table 2**, delayed gastric emptying was a GI condition frequently reported in AN and BN studies and delayed transit times in the small bowel (52, 56) and the colon (33, 56) were also reported. However, two studies found normal gastric emptying in AN (76) and BN (39) compared with controls and one study reported both delayed and rapid gastric emptying in BN (92).

*GI outcomes over time* were reported by 30 studies with a pre-post design. Different subjective GI symptoms were examined pre- and post-treatment in 15 studies. Overall, an improvement of most subjective GI symptoms was reported for all studies after treatment. The most frequently reported objective GI outcome for pre- and post-treatment was delayed gastric emptying (14 of 30 pre-post studies, 46.7%). Except for one study, delayed gastric emptying improved at least slightly upon treatment. In six of the pre-post studies with gastric emptying, medication was applied additionally. Medication administered to improve delayed gastric emptying included metoclopramide (in four studies) as well as erythromycin (39) and domperidone (95).

#### Overview of Study Outcomes at a Quantitative Level for Group 1—Subgroup Analysis

**Figure 3:**
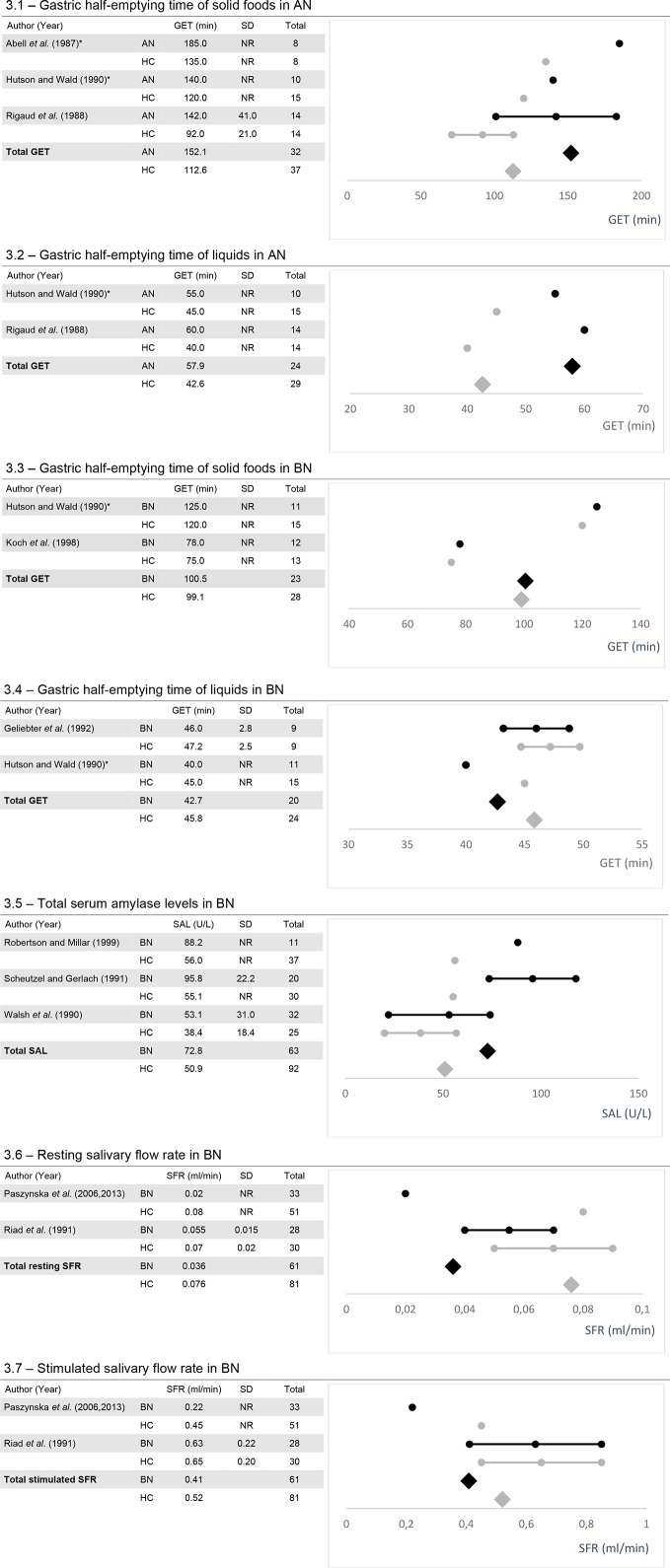
The figures show the mean values, standard deviation (SD) if reported and, calculated weighted total values for gastric half-emptying time (GET; 3.1-3.4), serum amylase levels (SAL; 3.5) and salivary flow rate (SFR; 3.6 and 3.7) along with the number of total participants (Total). AN, Anorexia nervosa; BN, Bulimia nervosa; HC, healthy controls; NR, Not reported. *Data were taken from figures.

Quantitative subgroup analyses for gastric half-emptying time, serum amylase levels of total amylase (including salivary and pancreatic amylase), and salivary flow rate are presented in **Figure 3**.

*For gastric half-emptying time*, five out of potentially 19 studies remained for subgroup analysis [for AN (24, 55, 79)]; for BN (47, 55, 60). Total gastric half-emptying time was delayed in AN compared to healthy controls for both liquids and, solid foods (**Figures 3.1** and **3.2**). In BN, gastric half-emptying time of solid foods was normal among considered studies whereas for liquids, it tended to be slightly more rapid in patients compared to healthy controls (**Figures 3.3** and **3.4**).

*Subgroup analysis of serum amylase levels* was conducted for three out of potentially five studies including patients with BN (82, 90, 102). Among all considered studies, total serum amylase was elevated in patients with BN in comparison to healthy controls (**Figure 3.5**).

*Subgroup analysis of salivary flow rate* was conducted from studies examining salivary flow rate in parotid glands. For this analysis, two out of five potential studies remained for analysis (73, 74, 78). Total resting salivary flow rate was reduced in BN patients versus healthy controls (**Figure 3.6**). Total stimulated salivary flow rate tended to be slightly reduced in BN with one study reporting values within the normal range and the other a reduced flow rate (**Figure 3.7**).

#### Group 2—Case Reports With ED Diagnosis

*GI measurements and outcomes* of group 2 are depicted in **Supplementary Material Table 2**. Diagnostic methods in group 2 differed from those used in group 1. The main subjective measurement was anamnesis/medical history which is usually taken on admission, while the use of questionnaires was rarely reported. Objective measurements were diverse and played a more important role. The most frequently reported methods were physical examination (68/62.4%), radiography (54/52.3%), computed tomography (CT) (44/40.4%), upper GI endoscopy (28/25.7%), dental/oral examination (11/10.1%), ultrasonography (10/9.2%), lower GI endoscopy (7/6.4%), and post-mortem autopsy (7/6.4%).

**Figure 4:**
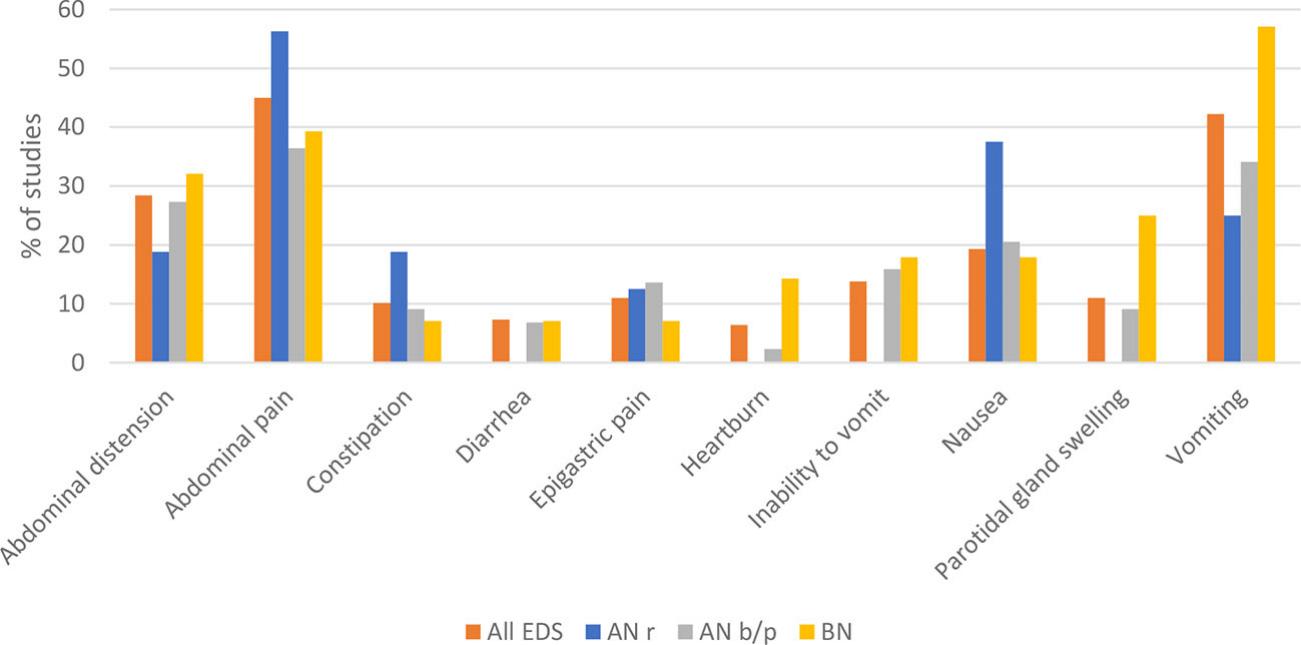
Subjectively reported gastrointestinal (GI) outcomes of case reports that were reported most frequently among all EDs are presented for eating disorders (ED), Anorexia nervosa (AN) differentiated for restrictive subtype (r) and binge/purge subtype (b/p), and Bulimia nervosa (BN). GI symptoms presented for ED are not the calculated sums of separate EDs shown in this figure. It is the summary of studies shown in table 1 of the supplements which assessed more than one ED. The data of the figure are presented below as sample size/% of studies reporting the symptom. ED: abdominal pain (49/45.0%), vomiting (46/42.2%), abdominal distension (31/28.4%), nausea (21/19.3%), inability to vomit (15/13.8%), epigastric pain (12/11.0%), parotid gland swelling (12/11.0%), constipation (11/10.1%), diarrhea (8/7.3%), and heartburn (7/6.4%). Subjective GI outcomes differed between EDs. The most frequent were in AN with restrictive subtype: abdominal pain (9/56.3%), nausea (6/37.5%), vomiting (4/25.0%), constipation (3/18.8%), abdominal distension (3/18.8%), and epigastric pain (2/12.5%); in AN with binge/purge subtype: abdominal pain (16/36.4%), vomiting (15/34.1%), abdominal distension (12/27.3%), nausea (9/20.5%), inability to vomit (7/15.9%), epigastric pain (6/13.6%), constipation (4/9.1%), parotid gland swelling (4/9.1%), diarrhea (3/6.8%), and heartburn (1/2.3%); in BN: vomiting (16/57.1%), abdominal pain (11/39.3%), abdominal distension (9/32.1%), parotid gland swelling (7/25.0%), inability to vomit (5/17.9%), nausea (5/17.9%), heartburn (4/14.3%), constipation (2/7.1%), diarrhea (2/7.1%), and epigastric pain (2/7.1%).

Subjectively reported GI problems of case reports are presented in **Figure 4** for studies with AN and BN patients. The most frequently reported symptoms were abdominal pain in both AN subtypes (AN restrictive: 9/56.3%; AN binge/purge: 16/36.4%) and vomiting in BN (15/34.1%).

An overview of the objective GI outcomes for EDs of group 2 is presented in **Table 3**. Most frequent objective outcomes among all EDs were gastric dilatation, gastric necrosis, gastric perforation, superior mesenteric artery syndrome, gastric wall ischemia, parotid gland hypertrophy, absence of bowel sounds, and duodenal dilatation. Differences between EDs could also be found and are presented in **Table 3**. Some GI problems often occurred together in the same case. The association of gastric dilatation with gastric necrosis and gastric perforation is worth mentioning in particular, as the two conditions hardly occurred without gastric dilatation.

**Table 3:** Group 2: Objective GI problems reported by case reports.

	ED		AN r		AN b/p		BN	
	n	%	n	%	n	%	n	%
Gastric dilatation	53	48.6	4	28.6	25	58.1	12	42.9
Gastric necrosis	13	11.9	0	0.0	7	16.3	2	7.1
Gastric perforation	11	10.1	1	7.1	8	18.6	1	3.6
Superior mesenteric artery syndrome	14	12.8	2	14.3	4	9.3	1	3.6
Gastric wall ischemia	11	10.1	1	7.1	5	11.6	2	7.1
P-arotid gland hypertrophy	13	11.9	0	0.0	5	11.6	7	25.0
Absence of bowel sounds	10	9.2	1	7.1	5	11.6	3	10.7
Duodenal dilatation	10	9.2	3	21.4	3	7.0	1	3.6
GI bleeding	8	7.3	2	14.3	2	4.6	2	7.1
Peritonism/Peritonitis	5	4.6	2	14.3	2	4.6	0	0.0
Submandibular gland hypertrophy	7	6.4	0	0.0	3	7.0	4	14.3

Legend: Objective GI outcomes which were reported most frequently are presented for all eating disorders together (ED), Anorexia nervosa with restrictive subtype (AN r), Anorexia nervosa with binge/purge subtype (AN b/p) and, Bulimia nervosa (BN). The number (n) and percentage (%) of studies reporting the specific GI outcome are shown. GI symptoms presented for ED are not the calculated sums of separate EDs shown in this figure. It is the summary of studies shown in **Supplementary Material Table 1** of the supplements which assessed more than one ED.

Lethal outcomes were reported in 16 studies with 17 (12.0% of all cases) deceased patients altogether, including one suicide (164). The highest mortality among case reports was found in the AN binge/purge subtype with lethal outcomes reported in 20.5% of studies reporting an AN binge/purge subtype (9 of 44) followed by 10.7% in studies reporting a BN (3 of 28). The association of gastric dilatation with lethal outcome was also higher in the AN binge/purge subtype than in BN (26.9% of AN binge/purge subtype cases with gastric dilatation were lethal vs. 16.7% in BN) and also when adjusted for higher frequency of gastric dilatation in AN binge/purge (AN binge/purge 16.5% and BN 8.0%).

#### Results of Risk of Bias Assessment

Risk of bias was assessed for all studies of group 1 according to OHAT criteria. Therefore, the 10 questions listed in the **Table 4** legend were applied to each study if considered appropriate, based on OHAT criteria and depending on study design. Study designs are summarized in the section “Summary of study characteristics” and reported individually in **Supplementary Material Table 1**. Across the studies, selection bias was rated very differently, with most studies showing probably low to probably high risk of bias. For confounding bias, 58% of studies were rated with a probably low risk of bias, whereas 32% showed a probably high risk of bias. The performance bias was mostly probably low to low where applicable. The attrition/exclusion bias did well, with most of the studies showing low (45%) or a probably low (36%) risk of bias. Detection bias was mostly low (27%) or probably low (55%). This was similar for selective reporting bias which also scored mostly low (29%) or probably low (56%). For group 2, the risk of bias in studies which only comprised case reports was estimated to be high due to the nature of the study design.

**Table 4:** OHAT: Risk of bias assessment.

Author (year)	SB			CB	PB		A/EB	DB		SRB
	1	2	3	4	5	6	7	8	9	10
Abell et al. (24)	#	#	++	+	#	#	++	+	+	++
Abraham et al. (25)	#	#	++	+	#	#	++	+	-	-
Arii et al. (26)	- -	- -	#	#	#	- -	-	+	+	++
Benini et al. (27)	-	++	#	#	#	+	++	+	+	++
Benini et al. (28)	- -	++	#	#	#	- -	+	+	+	++
Bluemel et al. (29)	- -	-	#	#	#	- -	++	+	++	++
Bozzato et al. (30)	#	#	+	+	#	#	++	++	++	+
Chami et al. (31)	NA	NA	#	#	#	NA	+	+	+	++
Chiarioni et al. (32)	- -	-	#	#	#	- -	++	++	++	+
Chun et al. (33)	#	#	-	-	#	#	+	+	-	-
Coddington and Bruch (34)	#	#	-	-	#	#	-	+	+	+
Cremonini et al. (9)	#	#	+	+	#	#	-	+	-	+
Crowell et al. (35)	#	#	- -	+	#	#	+	+	+	+
Cuntz et al. (36)	- -	-	#	#	#	-	++	++	++	++
DeJong et al. (37)	#	#	+	+	#	#	NA	-	+	+
Devlin et al. (38)	#	#	++	+	#	#	+	++	+	++
Devlin et al. (39)	#	#	++	++	#	#	+	+	+	+
Diamanti et al. (40)	#	#	- -	-	#	#	++	++	+	+
Domstad et al. (41)	#	#	-	-	#	#	+	++	-	+
Dooley-Hash et al. (42)	#	#	-	-	#	#	- -	- -	- -	- -
Dubois et al. (43)	#	#	++	+	#	#	+	++	+	-
Fernandez-Aranda et al. (44)	#	#	+	++	#	#	++	-	++	+
Fisher et al. (45)	#	#	-	+	#	#	++	+	NA	-
Garcia Aroca et al. (46)	*++*	-	#	#	#	-	*++*	*++*	*++*	*++*
Geliebter et al. (47)	#	#	-	+	#	#	+	++	++	+
Geliebter et al. (48)	#	#	+	++	#	#	++	++	+	++
Gowen et al. (49)	#	#	+	+	#	#	++	++	++	++
Heruc et al. (50)	#	#	++	+	#	#	++	++	+	++
Hill et al. (51)	#	#	+	-	#	#	+	++	-	- -
Hirakawa et al. (52)	#	#	+	+	#	#	++	++	+	+
Holmes et al. (53)	#	#	-	+	#	#	++	+	+	-
Hotta et al. (54)	NA	NA	#	#	#	NA	++	++	+	+
Hutson and Wald (55)	#	#	-	+	#	#	++	++	-	+
Kamal et al. (56)	#	#	-	+	#	#	++	+	+	+
Keel et al. (57)	#	#	++	- -	#	#	++	++	++	- -
Kinzl et al. (58)	#	#	+	+	#	#	*++*	*++*	*++*	*++*
Kiss et al. (59)	#	#	NA	+	#	#	++	++	++	- -
Koch et al. (60)	#	#	++	++	#	#	++	+	+	+
Lee et al. (61)	#	#	++	+	#	#	+	+	+	+
Levy et al. (62)	#	#	++	+	#	#	+	+	+	+
Lobera et al. (63)	#	#	-	+	#	#	+	+	+	+
Mack et al. (11)	NA	NA	#	#	#	NA	-	++	+	++
Mattheus et al. (64)	#	#	NA	+	#	#	+	++	++	++
McCallum et al. (65)	#	#	-	+	#	#	-	-	-	- -
Metzger et al. (66)	#	#	++	+	#	#	+	+	++	+
Mond et al. (67)	#	#	++	+	#	#	+	-	-	+
Nakai et al. (68)	#	#	+	+	#	#	++	-	+	+
Nickl et al. (69)	#	#	+	+	#	#	++	+	+	+
Ogawa et al. (70)	#	#	-	-	#	#	++	+	+	+
Ogren et al. (71)	#	#	NA	+	#	#	+	++	++	- -
Palla and Litt (72)	#	#	+	-	#	#	+	+	+	+
Paszynska et al. (73)	#	#	-	-	#	#	-	+	+	+
Paszynska et al. (74)	-	-	#	#	#	-	+	+	+	++
Peat et al. (75)	#	#	++	++	#	#	+	+	-	- -
Perez et al. (76)	- -	-	#	#	#	-	++	+	+	+
Price et al. (77)	+	-	#	#	#	-	++	+	+	+
Riad et al. (78)	#	#	-	-	#	#	-	+	-	+
Rigaud et al. (79)	- -	-	#	#	#	-	+	-	+	+
Roberts and Li (80)	#	#	- -	+	#	#	+	-	-	+
Roberts et al. (81)	#	#	- -	-	#	#	+	+	+	+
Robertson and Millar (82)	#	#	-	-	#	#	++	+	-	-
Robinson (83)	#	#	-	-	#	#	-	-	+	+
Rothstein (84)	NA	NA	#	#	#	NA	++	++	++	++
Rytomaa et al. (85)	#	#	-	-	#	#	++	+	-	++
Saleh and Lebwohl (86)	- -	-	#	#	#	-	+	+	+	+
Salvioli et al. (87)	NA	NA	#	#	#	NA	- -	++	+	++
Santos et al. (88)	#	#	+	-	#	#	+	+	+	+
Satoh and Yoshihara (89)	#	#	+	+	#	#	+	+	+	+
Scheutzel and Gerlach (90)	#	#	+	+	#	#	+	++	+	++
Sherman et al. (91)	#	#	-	-	#	#	-	+	+	+
Shih et al. (92)	#	#	+	+	#	#	++	+	+	+
Sileri et al. (93)	#	#	+	+	#	#	++	+	-	++
Silverstone and Russell (94)	#	#	+	+	#	#	++	++	++	++
Stacher et al. (95)	-	-	#	#	#	-	++	-	+	+
Szmukler et al. (96)	-	-	#	#	#	-	-	+	+	+
Thornton et al. (97)	#	#	++	++	#	#	-	+	+	+
Thornton et al. (98)	#	#	++	++	#	#	-	+	+	++
Tylenda et al. (99)	#	#	+	-	#	#	+	+	-	+
Valena et al. (100)	#	#	-	+	#	#	+	+	+	+
Waldholtz and Andersen (101)	NA	NA	#	#	#	NA	++	++	+	+
Walsh et al. (102)	#	#	-	-	#	#	+	++	+	+
Walsh et al. (103)	#	#	+	+	#	#	++	++	++	+
Winstead and Willard (14)	#	#	- -	-	#	#	-	-	-	+
Wockel et al. (104)	#	#	-	+	#	#	+	+	-	++
Wolff et al. (105)	#	#	- -	- -	#	#	++	+	++	++
Zimmerli et al. (106)	#	#	+	+	#	#	++	+	+	+

^++^definitely low risk of bias; ^+^probably low risk of bias; ^ -^probably high risk of bias; (also if NR); ^ - -^definitely high risk of bias;

^#^This criterion is not appropriate for this study type according to OHAT; NA, Not applicable.

Selection bias (SB):

1. Was administered dose or exposure level adequately randomized?

2. Was allocation of study groups adequately concealed?

3. Did selection of study participants result in appropriate comparison groups?

Confounding bias (CB):

4. Did the study design or analysis account for important confounding and modifying variables?

Performance bias (PB):

5. Were experimental conditions identical across study groups?

6. Were the research personnel and human subjects blinded to the study group during the study?

Attrition/Exclusion bias (A/EB):

7. Were the outcome data complete without attrition or exclusion from analysis?

Detection bias (DB):

8. Can we be confident in the exposure characterization?

9. Can we be confident in the outcome assessment?

Selective reporting bias (SRB):

10. Were all measured outcomes reported?

## Discussion

In this review, we systematically analyzed GI symptoms and complications across AN, BN, and BED. GI problems showed overlaps among the analyzed studies between the different EDs. Frequent overlapping GI symptoms were nausea, abdominal pain, and meteorism. The reporting of vomiting also ranked high among all EDs. However, most studies did not differentiate between involuntary and ED-dependent vomiting. Apart from general symptoms that were frequent in all EDs, more specific patterns were also found.

Constipation occurred frequently in EDs but was a frequent symptom for AN in particular, most likely due to chronic food restriction. Another symptom occurring frequently in AN was abdominal fullness. On the one hand, abdominal fullness can be used as a reason for not being able to eat (216). On the other hand, this fits with objectively measured delayed gastric emptying time reported in most studies at a qualitative level and supported by our quantitative analyses. In addition, several studies also reported delayed transit times for other regions of the GI tract such as the small bowel (52, 56) and the colon (32, 33). Overall, gastric emptying time can improve after mid- to long-term rehabilitation but not after short-term refeeding (27, 79). Thus, the delayed gastric emptying time together with the experienced abdominal fullness might be a challenging GI problem in AN, making the introduction and acceptance of normal portion sizes of meals difficult.

Similarly, other pre-post design studies overall reported an improvement in most GI conditions. Interestingly, Chami et al. (31) reported that GI symptoms improved, but not significantly if controlled for the mediating effects of depression (31). Mack et al. (11) found a difference between upper and lower GI symptoms before and after treatment with lower GI symptoms mostly improving and upper GI symptoms tending to persist (11). Perez et al. (76) reported a decrease in irritable bowel syndrome prevalence, in addition to the general improvement of GI symptoms after treatment (76).

In BN, and to a limited extent also in AN of the binge/purge subtype, salivary gland pathologies such as parotid, submandibular or minor salivary gland hypertrophy were described in several case reports. An explanation for this is that eating large amounts of food in a short time with subsequent vomiting are stimuli for enhanced saliva production and thus enlargement of the salivary glands (58, 82, 102). Group 1 studies that measured salivary gland sizes with technical methods such as ultrasonography, overall supported the subjective findings (30, 66), except for one study with a small sample size (n=5) (77). Enhanced saliva production was also reported among studies of group 1 which was often measured as elevated total or salivary amylase in the serum. This hyperamylasemia is also supported by our quantitative analysis. In this analysis, we compared total, instead of salivary serum amylase levels because one of our included studies (82) only reported total and pancreatic serum amylase because measurement of salivary serum amylase was not feasible due to the facilities available. Analyzing total serum amylase, which is assumed to be the sum of salivary and pancreatic serum amylase (82), was possible, as pancreatic serum amylase was measured in all included studies and not found to be elevated. Thus, elevated total serum amylase resulting from pancreatic damage can be ruled out. Although salivary gland hypertrophies and elevated total serum or salivary amylase are reported, BN patients showed reduced resting salivary flow rates. Several studies tried to find an explanation for this circumstance.

One hypothesis is that frequent vomiting leads to dehydration, which leads to reduced saliva production during resting (217). Other studies postulate that the frequent use of antidepressants in BN patients, of which some have proven anticholinergic effects (218–220), might cause a decrease in salivary gland output (74). However, the downregulation of the saliva production could also be seen as a mechanism to compensate for the high amounts of saliva required during purging episodes.

Another cluster of GI problems in BN is also provoked by frequent vomiting. These were heartburn as a subjective symptom, but also dental erosion, esophagitis, caries and gastroesophageal reflux (GER) which were examined objectively.

Although case reports as one-patient studies should be regarded with more caution, they play an important role in the overview of GI problems in EDs by highlighting rare life-threatening complications that would be difficult to examine in studies with larger sample sizes. Subjective GI symptoms in group 2 appeared to be similar to those reported in group 1. Nevertheless, objective GI disturbances in particular, extended the range of important GI problems by including a variety of serious complications which have also been discussed recently by Norris et al. (221). A frequently reported complication was gastric dilatation due to superior mesenteric artery syndrome. These patients usually presented at an emergency ward after a binge eating episode (AN with binge/purge subtype, followed by BN), often complaining about nausea and the inability to vomit, as well as abdominal pain, abdominal fullness and showing visible abdominal distension. The situation of a patient with gastric dilatation becomes even more threatening if the stomach becomes necrotic or perforated.

Besides binge eating behavior, emaciation also predisposes for the development of superior mesenteric artery syndrome, as a lack of intestinal fat lowers the aortomesenteric angle which can lead to obstruction of the upper part of the duodenum (108). The combination of these two risk factors in particular put patients with AN binge/purge subtype at risk of severe or fatal developments of superior mesenteric artery syndrome with gastric dilatation and its complications. Another contributing factor for the high lethal outcome in case reports with AN binge/purge subtype is that AN itself has a high mortality rate, which is 12 times higher than any other cause in women aged 15-24 years (150). Nevertheless, the results regarding lethal outcomes among our studies could also be due to reporting bias since a lethal case is more likely to be reported.

During the literature search, we encountered several strengths and limitations of this review which are discussed below. First, we would like to emphasize that the information of the percentage of studies that report a certain GI symptom per ED diagnosis (**Tables 2** and **3**, **Figures 2** and **4**) is limited due to the different GI symptoms/problems assessed by the single studies. Therefore, the percentages only represent the amount of studies among the included studies that reported a specific GI condition and must not be confused with prevalences.

Pre-post design studies were rare and heterogenous, making it impossible to extract specific patterns of GI problems that generally improved, persisted or worsened, especially for each ED separately. No data was available describing the development of GI problems in the time course of treatment.

Another limitation was the underrepresentation of certain groups, e.g. male patients with EDs. On the one hand, we did not find many studies with balanced sex ratios or some with even any male patients at all. On the other hand, studies as well as clinical experience show that men are indeed less affected by EDs (6), therefore it is more difficult to study the GI symptoms or any other characteristics of male EDs.

Another underrepresented group were patients with BED, as BED has only been considered a diagnosis since the publication of the DSM-5 in 2013. It was impossible to identify a certain GI pattern for BED due to the low number of studies. As obesity is a wide spread condition, BED should supposedly be as well. Presumably, the latter is rarely assessed in emergency wards due to low awareness. It is also possible that BED patients rarely develop acute complications, unlike other EDs, as BED patients develop more chronic complications due to obesity and metabolic syndrome. However, the status on GI disturbances in BED is not well examined and could be of further research interest, especially to better understand the development of the disorder.

Finally, studies were heterogenous with regard to their aims, methods, outcomes, and study designs. In particular, high-quality randomized controlled trials, which are the basis for meta-analytical approaches, were rare (n=4) and additionally, covered incomparable GI topics.

This finding highlights the importance and strength of this review to include a broad range of study designs to avoid selection bias. In order to illustrate the quality of the included studies, a risk of bias assessment was performed to help the reader estimate the quality of each individual study without the necessity to narrow down the broader picture due to exclusion of studies. In addition, observational and experimental studies also contribute valuable aspects to the state of knowledge, and RCTs are not always ethical and/or feasible as is the case in the field of EDs and GI problems. We therefore conducted quantitative analyses with controlled trials where possible. The limitations of these analyses were different applied techniques or reported parameters. However, these quantitative analyses are very valuable to support the qualitative level findings.

Although not a topic of this review but worth mentioning is the prevalence of misdiagnosis of GI related diseases in ED that we encountered during our search process. These included Crohn’s disease, inflammatory bowel diseases, achalasia, celiac disease, and functional diseases such as irritable bowel syndrome and rumination syndrome. In some studies, ED diagnosis preceded the diagnosis of GI diseases whereas in others, the ED developed after GI disease diagnosis (222). In other cases, the two diseases coexisted which sometimes led to further complications in treatment (223). To deal with this in practice, we recommend that physicians such as gastroenterologists, who usually have more contact with patients with somatic GI diseases, utilize the short SCOFF questionnaire (224) for clinical assessment of patients in whom an ED is suspected. On the other hand, psychiatrists and psychotherapists in charge of treating EDs should be aware that GI problems are common in these patients, but to only screen for other possible GI diseases if symptom patterns are fitting. GI diseases are not necessarily more prevalent in ED than in the normal population e.g. the prevalence of celiac disease is not higher than in the control population (225).

In summary, this systematic research study presents an overview of the wide-ranging topic of GI disturbances in the EDs AN, BN, and BED. Problems in the GI tract are frequent in EDs and it is likely that they protract therapy, especially in AN. Many GI problems are linked to disordered eating and food intake behavior such as chronic food restriction, binge eating, vomiting, and the abuse of laxatives and, improve after treatment.

Finally, there are research gaps which warrant further research. To date, it is not completely clear which GI symptoms and complications occur in BED. It is unknown to which extent GI problems protract ED therapy, especially in AN, and how GI symptoms change during the time course of treatment. Understanding the underlying GI physiology and problems in the course of refeeding may help to identify critical periods of GI wellbeing, which could then be addressed more adequately.

## Data Availability Statement

All datasets generated for this study are included in the article/**Supplementary Material**.

## Author Contributions

IM, CR, and GS contributed to the conception and design of the study. CR, GS, and AW organized the database. CR and IM wrote the first draft of the manuscript. KG, AS, PE, and SZ wrote sections of the manuscript. All authors contributed to manuscript revision, and read and approved the submitted version.

## Funding

IM received a grant from the Ministry of Science Baden-Württemberg and the European Social Fund. The authors acknowledge support by the Deutsche Forschungsgemeinschaft and the Open Access Publishing Fund of Tübingen University.

## Conflict of Interest

The authors declare that the research was conducted in the absence of any commercial or financial relationships that could be construed as a potential conflict of interest.
