# Supplementary material for: Which Symptoms, Complaints and Complications of the Gastrointestinal Tract Occur in Patients With Eating Disorders? A Systematic Review and Quantitative Analysis
Source: Front Psychiatry. 2020 Apr 20;11:195. doi: 10.3389/fpsyt.2020.00195 (PMC7212454; doi:10.3389/fpsyt.2020.00195)
Supplement: Supplementary file 1 [file DataSheet_1.pdf]

Supplementary material

Supplement Table 1 – Group 1: Human Studies with ED diagnosis, excluding case reports

**Author (year)**	**Study type & follow-up**	**ED criteria**	**Characterization**			**GI-related measurements S+O**	**GI outcomes S+O**	**Intervention**
			n/m	Age (years)	BMI			
Studies with Anorexia nervosa, restrictive subtype								
Abell *et al.* (1987) (1)	OCS, NRNCT 4-15m follow-up of 5 patients	DSM-III	8/2	20	NR	O: GE, antral phasic pressure activity, gastric myoelectrical activity, dietary intake	O: pre c↑, antral motility↓, gastric electrical dysrhythmia → *post* GE & antral motility improved slightly, g.e.d continues	NRP
Arii *et al.* (1996)* (2)	RCT 12w follow-up	DSM-III-R	9/0	17.3	13.2	S: SCQ	S: *pre* abdominal fullness, hemorrhoids, constipation, diarrhea, fracture of pubic bone → *post* bowel movement rate↑ in LF group	LF, PT
Benini *et al.* (2004)* (3)	NRCT 4&22w follow-up of 17 patients	APA crit.	11/0	19.9	13.2	S: SCL-90, BSQ<br>O: GE	S: *pre* vomiting, nausea, abdominal fullness, decreased appetite, constipation, meteorism → *post* GI symptoms slightly improved<br>O: *pre* GE↑ → *post* GE improved	NRP, PT
Benini *et al.* (2010)* (4)	NRCT 4&22w follow-up of 17 patients	APA crit.	11/0	19.9	13.2	S: SCL-90, BSQ<br>O: esophageal manometry	S: *pre* heartburn, vomiting, regurgitation, dysphagia, abdominal pain, nausea, decreased appetite, constipation, meteorism → *post* gastric & colonic but not esophageal symptoms improved<br>O: *pre* LES pressure↑ → *post* LES pressure normalized	NRP, PT
Cuntz *et al.* (2013)* (5)	NRCT 2 follow-ups of patients (& 5 controls)	DSM-4	10/0	26.1	14.6	S: GIS, GIM	S: *pre* vomiting, heartburn, regurgitation, dysphagia, nausea, abdominal pain, abdominal fullness, abdominal distension, borborygmi, constipation, meteorism, diarrhea, urgency → *post* GI scores improved	NRP, PT
Heruc *et al.* (2018) (6)	OCS, NRNCT 1&2w follow-up	DSM-5<br>EDE-Q	22/0	15.9	NR	S: GISS-Q	S: *pre* meteorism, abdominal fullness, decreased appetite → *post* abdominal fullness, decreased satiety, and GI symptom scores but not meteorism improved	NRP
Holmes *et al.* (2016)* (7)	ONCS analysis 5 y retrospectively	DSM-IV-R<br>DSM-5	125/ NR (206/ 19[1])	NR (28[1])	NR (12.9[1])	S: SCQ	S: dysphagia	NA
Hotta *et al.* (2009) (8)	NRNCT 26d follow-up	DSM-IV	5/0	26.4	13	S: SCQ	S: *pre* epigastric pain, abdominal pain, abdominal fullness, decreased appetite, constipation → *post* abd. pain & fullness normalized, appetite improved	NRP, PT, ghrelin infusion
Lee *et al.* (2012)* (9)	ONCS no follow up	DSM-IV	88/NR	NR (20.6[1])	NR (15.4[1])	S: SCQ, SCL-90	S: epigastric pain, nausea, meteorism	NA

Supplement Table 1 – Group 1: Human Studies with ED diagnosis, excluding case reports

Author (year)	Study type & follow-up	ED criteria	Characterization			GI-related measurements S+O	GI outcomes S+O	Intervention
			n/m	Age (years)	BMI			
Mack *et al.* (2016)* (10)	NRCT follow-up after weight gain	NR	39/0	NR (23.8[1])	NR (15.3[1])	S: G-Q	S: *pre* heartburn, regurgitation, abdominal fullness, abdominal distension, meteorism → *post* lower GI symptoms improved, upper GI symptoms continued	NRP
Mattheus *et al*. (2019) (11)	ONCS no follow-up	ICD-10	253/7	NR (15.1[1])	NR (15.3[1])	S: SCQ	S: constipation	NA
Nakai *et al.* (2017)* (12)	OCS 85.2m follow-up (recruitment: retrospect. chart review)	DSM-5	63/0	NR (20.1[2])	NR (14.6[2])	S: SCQ	S: abdominal pain, abdominal fullness, meteorism	NA
Ogawa *et al.* (2004)* (13)	OCS no follow-up	DSM-IV	2/0	22	NR	S: SCQ<br>O: electrogastrography	S: heartburn, epigastric pain, nausea<br>O: gastric electrical dysrhythmia	NR
Palla & Litt (1988)* (14)	ONCS no follow-up	DSM-III	32/NR	15.3	NR	O: dental/oral examination	S: salivary gland swelling, heartburn, regurgitation, abdominal fullness, constipation, meteorism<br>O: esophagitis	NA
Roberts & Li (1987)* (15)	ONCS no follow-up	NR	6/0	28	NR	O: dental/oral examination	O: dental erosion	NA
Santos *et al.* (2016) (16)	OCS no follow-up	NR	8/1	25.9	19.2	O: videofluoroscopy for swallowing pattern	O: Swallowing pattern ↔	NA
Scheutzel & Gerlach (1991)* (17)	OCS no follow-up	DSM-III-R	12/0	22	NR	O: salivary & serum amylase, dental/oral examination, salivary flow rate	S: salivary gland swelling<br>O: hyperamylasemia	NR
Sileri *et al.* (2014)* (18)	OCS no follow-up	AMA crit.	NR/NR (85/2[1])	NR (28[1])	NR (16[1])	S: WCS, AOD-S, FISI	S: constipation, faecal incontinence	NA
Szmukler *et al.* (1990)* (19)	ONCS, NRNCT 4-15m follow-up of 5 patients	DSM-III-R	18/0	22.8	14.3	O: GE	O: *pre* GE↑ → *post* GE improved with weight gain	LF, NRP
Waldholtz & Andersen (1990)* (20)	NRCT 12w follow-up	AMA crit.	11/0	NR	NR	S: SCQ, GISS	S: *pre* heartburn, vomiting, dysphagia, eructation, abdominal pain, nausea, decreased appetite, borborygmi, cramps, constipation, diarrhea, meteorism → *post* all GI symptoms except eructation improved	NRP, ghrelin infusion
Winstead & Willard (2006)* (21)	OCS 1y follow-up	SCQ	34/NR[2] (63/6[1])	NR (27.6[1])	NR	S: SCQ	S: heartburn	NA

Supplement Table 1 – Group 1: Human Studies with ED diagnosis, excluding case reports

Author (year)	Study type & follow-up	ED criteria	Characterization			GI-related measurements S+O	GI outcomes S+O	Intervention
			n/m	Age (years)	BMI			
Studies with Anorexia nervosa, binge/purge subtype								
Arii *et al.* (1996)* (2)	RCT<br>12w follow-up	DSM-III-R	2/0	22.3	14.9	S: SCQ	S: abdominal fullness, hemorrhoids,<br>constipation, diarrhea, fracture of pubic bone	LF, NRP
Benini *et al.* (2004)* (3)	NRCT<br>4&22w follow-up	APA crit.	12/0	25.4	15.5	S: SCL-90, BSQ<br>O: GE	S: vomiting, nausea, abdominal fullness, decreased appetite, constipation, meteorism<br>O: GE↑	NR
Benini *et al.* (2010)* (4)	NRCT<br>4&22w follow-up	APA crit.	12/0	25.4	15.5	S: SCL-90, BSQ<br>Esophageal manometry	S: heartburn, vomiting, regurgitation, dysphagia, abdominal pain, nausea, decreased appetite, constipation, meteorism<br>O: LES pressure↔	NR
Cuntz *et al.* (2013)* (5)	NRCT<br>2 follow-ups of patients (& of 5 controls)	DSM-IV	13/1	24.7	15.5	S: GIS, GIM	S: *pre* vomiting, heartburn, regurgitation, dysphagia, nausea, abdominal pain, abdominal fullness, abdominal distension, borborygmi, constipation, meteorism, diarrhea, urgency → *post* GI scores improved	NR
Holmes *et al.* (2016)* (7)	ONCS<br>analysis 5y retrospect.	DSM-IV-R<br>DSM-5	81/NR<br>(206/ 19[1])	NR<br>(28[1])	NR<br>(12.9[1])	S: SCQ	S: dysphagia	NA
Lee *et al.* (2012)* (9)	ONCS<br>no follow up	DSM-IV	53/NR	NR<br>(20.6[1])	NR<br>(15.4[1])	S: SCQ, SCL-90	S: epigastric pain, nausea, meteorism	NA
Mack *et al.* (2016)* (10)	NRCT<br>follow-up after weight gain	NR	16/0	NR<br>(23.8[1])	NR<br>(15.3[1])	S: G-Q	S: heartburn, regurgitation, abdominal fullness, abdominal distension,<br>meteorism	NRP
Mattheus *et al.* (2019) (11)	ONCS<br>no follow-up	ICD-10	32/1	NR<br>(15.1[1])	NR<br>(15.3[1])	S: SCQ	S: constipation	NA
Nakai *et al.* (2017)* (12)	OCS<br>85.2m follow-up<br>(recruitment: retrospect. chart review)	DSM-5	45/0	NR<br>(20.1[2])	NR<br>(14.6[2])	S: SCQ	S: abdominal pain, abdominal fullness,<br>meteorism	NA
Ogawa *et al.* (2004)* (13)	OCS<br>no follow-up	DSM-IV	13/0	NR	NR	S: SCQ<br>O: electrogastrography	S: heartburn, epigastric pain, nausea<br>O: gastric electrical dysrhythmia	NR
Palla & Litt (1988)* (14)	ONCS<br>no follow-up	DSM-III	15/NR	15.7	NR	O: dental/oral examination	S: salivary gland swelling, heartburn, regurgitation, abdominal fullness,<br>constipation, meteorism<br>O: esophagitis	NR
Price *et al.* (2008) (22)	NRCT<br>2 m follow-up	NR	3/0	22.6	17.3	O: PE on salivary glands, MRI of parotid glands	O: enlarged salivary glands palpated, no salivary gland hypertrophy in MRI	NRP

Supplement Table 1 – Group 1: Human Studies with ED diagnosis, excluding case reports

Author (year)	Study type & follow-up	ED criteria	Characterization			GI-related measurements S+O	GI outcomes S+O	Intervention
			n/m	Age (years)	BMI			
Roberts & Li (1987)* (15)	ONCS no follow-up	NR	11/0	NR	NR	O: dental/oral examination	O: dental erosion	NR
Scheutzel & Gerlach (1991)* (17)	OCS no follow-up	DSM-III-R	13/1	25.6	NR	O: salivary & serum amylase, dental/oral examination, salivary flow rate	S: salivary gland swelling<br>O: hyperamylasemia	NR
Sileri *et al.* (2014)* (18)	OCS no follow-up	AMA crit.	NR/NR (85/2[1])	NR (28[1])	NR (16[1])	S: WCS, AOD-S, FISI	S: constipation, faecal incontinence	NA
Szmukler *et al.* (1990)* (19)	ONCS, NRNCT 4-15m follow-up	DSM-III-R	2/0	NR	15	O: GE	O: *pre* GE↑ → *post* GE improved with weight gain	LF, NRP
Waldholtz & Andersen (1990)* (20)	NRCT 12w follow-up	AMA crit.	5/0	NR	NR	S: SCQ, GISS	S: *pre* heartburn, vomiting, dysphagia, eructation, abdominal pain, nausea, decreased appetite, borborygmi, cramps, constipation, meteorism, diarrhea → *post* all GI symptoms except eructation improved	NRP, ghrelin infusion
Winstead & Willard (2006)* (21)	OCS 1y follow-up	SCQ	34/NR[2] (63/6[1])	NR (27.6[1])	NR	S: SCQ	S: heartburn	NA
Wolff *et al.* (1968)* (23)	OONCS	NR	5/0	NR (35.1[1])	19.3	S: SCQ	S: vomiting, nausea, constipation	NA

Studies with Anorexia nervosa, unclear/not reported subtype

Author (year)	Study type & follow-up	ED criteria	n/m	Age (years)	BMI	GI-related measurements S+O	GI outcomes S+O	Intervention
Abraham *et al.* (2012)* (24)	ONCS no follow-up	DSM-IV	84/0	23.6	15.6	S: SCQ, ROME II	S: fulfilled IBS crit., abdominal distension, meteorism	NA
Bluemel *et al.* (2017) (25)	NRCT 112 (69-161) d follow-up	DSM-IV	24/0	23	14.4	S: SCQ, short-form LDQ, GCSI<br>O: GE	S: *pre* abdominal fullness, decreased appetite → *post* abd. fullness improved<br>O: *pre* GE↑ → *post* GE improved	NRP, PT
Bozzato *et al.* (2008)* (26)	OCS no follow-up	DSM-IV	7/0	28.3	16.3	O: salivary gland biometry	O: salivary gland hypertrophy	NA
Chiarioni *et al.* (2000) (27)	NRCT 4w follow-up	AMA crit.	12/0	23.7	13.1	O: colonic transit time, anorectal manometry	S: constipation<br>O: *pre* colonic transit time↑, threshold for urge to defecate↑, resting anal pressure↓ → *post* colonic transit times normalized, threshold for urge to defecate and resting anal pressure did not	NRP, PT
Chun *et al.* (1997) (28)	OCS no follow-up	APA crit.	13/0	27	NR	S: SCQ<br>O: colonic transit time, anorectal manometry	S: constipation<br>O: GE↑	NR
Coddington & Bruch (1970) (29)	OCS no follow-up	NR	3/0	16	NR	O: gastric perceptivity	O: gastric perceptivity↓	NA

Supplement Table 1 – Group 1: Human Studies with ED diagnosis, excluding case reports

Author (year)	Study type & follow-up	ED criteria	Characterization			GI-related measurements S+O	GI outcomes S+O	Intervention
			n/m	Age (years)	BMI			
Diamanti *et al.* (2003)* (30)	OCS<br>no follow-up	DSM-IV	28/0	15	15.2	S: SCQ<br>O: GE, electrogastrography	S: epigastric pain, nausea, abdominal fullness<br>O: GE↑, gastric electrical dysrhythmia	NR
Domstad *et al.* (1987) (31)	ONCS, NRNCT<br>follow-up after treatment	DSM-III	26/3	21.4	NR	O: GE	S: vomiting, epigastric pain, nausea, abdominal fullness, meteorism<br>O: *pre* GE↑&↓ → *post* GE improved in some patients with MCP	MCP
Dooley-Hash *et al.* (2013)* (32)	OCS<br>no follow up	Based on SCOFF	NR (284/ 63[1])	NR	NR	S: SCQ	S: Vomiting, hematemesis, abdominal pain, nausea, constipation, diarrhea, rectal bleeding	NA
Dubois *et al.* (1979) (33)	OCS, NRNCT<br>4-10w follow-up	NR	15/0	24	NR	O: GE, pHmetry	O: *pre* GE↑, H^+ output↓ → *post* GE tended to improve	NRP, PT
Fernandez-Aranda *et al.* (2016 (34)	OCS<br>no follow-up	DSM-5	64/0	24	17.4	O: taste strips	O: Taste capacity across patients ↔	NA
Fisher *et al.* (2014)* (35)	OCS<br>no follow up	DSM-5	98/14	15.6	NR	S: SCQ	S: GI symptoms not specified	NA
Garcia Aroca *et al.* (2001) (36)	RNCT<br>no follow-up	NR	18/1	17.1	NR	O: antroduodenal manometry, pHmetry	S: vomiting, abdominal pain, constipation<br>O: *pre* gastroesophageal reflux, altered gastric motility → *post* erythromycin > cisapride improved GE	Cisapride Erythromycin
Hill *et al.* (1999)* (37)	OCS<br>no follow-up	DSM-IV	11/2	NR	NR	O: serum Helicobacter pylori IgG antibody	S: GI symptoms not specified<br>O: Helicobacter pylori infection↔	NR
Hirakawa *et al.* (1990) (38)	OCS<br>no follow-up	Modified Feigner's crit.	9/1	19	NR	O: small bowel transit time	O: small bowel transit time↑	NA
Hutson & Wald (1990)* (39)	OCS<br>no follow-up	DSM-III-R	10/1	28	NR	S: SCQ<br>O: GE	S: nausea, abdominal pain, decreased appetite, meteorism<br>O: GE↑	NR
Kamal *et al.* (1991)* (40)	OCS<br>no follow-up	DSM-III	10/1	26.2	15.1	S: BSQ<br>O: Small bowel & whole gut transit time	S: nausea, abdominal pain, constipation, meteorism<br>O: small bowel transit time↑, whole-gut transit time↑	NR
Kinzl *et al.* (1993)* (41)	OCS<br>no follow-up	DSM-III-R	NR	NR	17.2	O: salivary & serum amylase, dental/oral examination	S: salivary gland swelling, vomiting<br>O: hyperamylasemia	NR
McCallum *et al.* (1985) (42)	OCS<br>no follow-up	DSM-III	16/0	20	NR	S: SCQ<br>O: GE	S: vomiting, heartburn, epigastric pain, eructation, nausea, abdominal pain, decreased appetite, constipation, meteorism<br>O: *pre* GE↑ → *post* MCP improved GE	MCP

Supplement Table 1 – Group 1: Human Studies with ED diagnosis, excluding case reports

Author (year)	Study type & follow-up	ED criteria	Characterization			GI-related measurements S+O	GI outcomes S+O	Intervention
			n/m	Age (years)	BMI			
Perez *et al.* (2013) (43)	NRCT<br>14w follow-up	NR	16/0	15.5	17.3	S: CSI, ROME III<br>O: GE, gastric accomodation	S: *pre* nausea, abdominal pain, constipation, meteorism, diarrhea, fulfilled IBS crit. → *post* GI symptoms improved, IBS prevalence decreased<br>O: *pre* GE↔, postprandial antral diameter↓ → *post* p.p. antral diameter improved	NRP
Rigaud *et al.* (1988) (44)	NRCT<br>4-8w follow-up	DSM-III	14/1	26.7	NR	S: SCQ<br>O: GE	S: *pre* vomiting, epigastric pain, eructation,<br>nausea, abdominal pain, abdominal fullness, constipation → *post* GI symptoms normalized in most patients, correlating with improved GE for nausea, vomiting & fullness<br>O: *pre* GE↑ → *post* GE improved in patients with adequate weight gain	NRP
Robinson (1989)* (45)	OCS<br>follow-up of 8 patients after normal weight was reached	NR	22/NR	NR	NR	S: SCQ<br>O: GE	S: nausea, abdominal fullness, decreased satiety<br>O: GE↑	NR
Saleh & Lebwohl (1980) (46)	OCS, NRNCT<br>weekly follow-up during 1m	NR	7/2	28.3	14.9	O: GE	S: *pre* vomiting, epigastric pain, eructation,<br>decreased appetite, constipation, diarrhea → *post* overall GI symptoms improved<br>O: *pre* GE↑ → GE improved	MCP
Salvioli *et al.* (2013)* (47)	NRNCT<br>follow-up at discharge & 1 & 6 m after	DSM-IV-R	39/NR<br>(48/7[1])	NR	NR	S: WCS, EGI	S: *pre* heartburn, epigastric pain, dysphagia, regurgitation, abdominal pain, nausea, abdominal fullness, fulfilled IBS crit., decreased appetite, abdominal distension → *post* GI symptoms only improved in patients with normal hypochondriasis, hysteria & depression scores	NRP, PT
Satoh & Yoshihara (2004)* (48)	OCS<br>no follow-up	NR	31/NR	25.7	NR	O: parotid gland biopsy	O: hyperamylasemia, alteration of parotidal gland cells	NA
Sherman *et al.* (1993)* (49)	OCS<br>no follow-up	„according to established crit.“	23/0	NR	NR	O: serum Helicobacter pylori IgG antibody	S: vomiting, heartburn, eructation, abdominal pain, GI bleeding, constipation, diarrhea<br>O: Helicobacter pylori infection↔	NR
Silverstone & Russell (1967) (50)	OCS, NRNCT<br>follow-up of 5 patients after weight gain	NR	8/1	23	NR	O: gastric activity	O: *pre* gastric activity↔ → *post* gastric activity decreased below normal	NRP
Stacher *et al.* (1986) (51)	OCS, RCT<br>no follow-up	DSM-III<br>Feighner's crit.	NR	23.1	NR	O: GE, esophageal manometry, esophageal motor activity	S: vomiting, heartburn, epigastric pain, regurgitation, eructation, dysphagia,<br>abdominal fullness, constipation, meteorism<br>O: *pre* GE↑, gastroesophageal reflux, esophagitis,<br>UES↑, achalasia,<br>disordered esophageal motor activity → *post* domperidone improved delayed GE	NRP, PT, Domperi-done

Supplement Table 1 – Group 1: Human Studies with ED diagnosis, excluding case reports

Author (year)	Study type & follow-up	ED criteria	Characterization			GI-related measurements S+O	GI outcomes S+O	Intervention
			n/m	Age (years)	BMI			
Wockel *et al.* (2007)* (52)	OCS<br>no follow-up	NR	9/0	17.9	16.8	O: papilla counting, taste strips	O: amount of papillas on tongue↓	NA
Studies with Bulimia nervosa								
Abraham *et al.* (2012)* (24)	ONCS<br>no follow-up	DSM-IV	33/0	23.7	22.5	S: SCQ, ROME II	S. fulfilled IBS crit., abdominal distension, meteorism	NA
Bozzato *et al.* (2008)* (26)	OCS<br>no follow-up	DSM-IV	18/0	23.9	20.1	O: Salivary gland biometry	O: salivary gland hypertrophy	NA
Chami *et al.* (1995) (53)	NRCT<br>6w follow-up	AMA	43/6	23.3	22.7	S: GISS	S: *pre* heartburn, dysphagia, eructation,<br>abdominal pain, nausea, decreased appetite, borborygmi, constipation, meteorism, diarrhea<br>→ *post* GI sypmtoms improved (mediating effects of depression)	NRP, PT
DeJong *et al.* (2011) (54)	ONCS<br>no follow up	DSM-IV	51/NR<br>(64/4[1])	NR	NR<br>(23.4[1])	S: IBS-Q	S: vomiting, fulfilled IBS crit.	NA
Devlin *et al.* (1997) (55)	OCS, NRNCT<br>4-25w follow-up	DSM-III-R	8/0	26.9	22.3	O: GE	O: *pre* GE↑ → *post* GE did not improve	NRP
Devlin *et al.* (2012) (56)	OCS, RCT<br>6 w follow-up	NR	32/0	24	22.4	O: GE	S: vomiting<br>O: *pre* GE↔ → *post* erythromycin accelerated GE	Erythromycin
Diamanti *et al.* (2003)* (30)	OCS<br>no follow-up	DSM-IV	NR	17.8	20	S: SCQ<br>O: GE, electrogastrography	S: epigastric pain, nausea, abdominal fullness<br>O: GE↑, gastric electrical dysrhythmia	NR
Dooley-Hash *et al.* (2013)* (32)	OCS<br>no follow up	Based on SCOFF	NR<br>(284/63[1])	NR	NR	S: SCQ	S: vomiting, hematemesis, abdominal pain, nausea, constipation, diarrhea, rectal bleeding	NA
Fisher *et al.* (2014)* (35)	OCS<br>no follow up	DSM-5	66/4	16.5	NR	S: SCQ	S: GI symptoms not specified	NA
Geliebter *et al.* (1992) (57)	OCS<br>no follow-up	DSM-III-R	9/0	24.9	22.4	O: GE, gastric capacity	S: vomiting, decreased satiety<br>O: GE↑, stomach capacity↑	NA
Hill *et al.* (1999)* (37)	OCS<br>no follow-up	DSM-IV	4/0	NR	NR	O: serum Helicobacter pylori IgG antibody	S: GI symptoms not specified<br>O: Helicobacter pylori infection↔	NR
Hutson & Wald (1990)* (39)	OCS<br>no follow-up	DSM-III-R	11/0	27	NR	S: SCQ<br>O: GE	S: nausea, abdominal pain, decreased appetite, meteorism<br>O: GE↑	NR
Kamal *et al.* (1991)* (40)	OCS<br>no follow-up	DSM-III	18/3	27.3	22.9	S: BSQ<br>O: small bowel & whole gut transit time	S: nausea, abdominal pain, constipation, meteorism<br>O: small bowel transit time↑, whole-gut transit time↑	NR

Supplement Table 1 – Group 1: Human Studies with ED diagnosis, excluding case reports

Author (year)	Study type & follow-up	ED criteria	Characterization			GI-related measurements S+O	GI outcomes S+O	Intervention
			n/m	Age (years)	BMI			
Keel *et al.* (2007) (58)	OCS no follow-up	DSM-IV EDE-Q	37/0	21.1	22.3	S: SCQ	S: epigastric pain, vomiting, abdominal pain, nausea, abdominal fullness, decreased satiety	NA
Kinzl *et al.* (1993)* (41)	OCS no follow-up	DSM-III-R	NR	NR	NR	O: salivary & serum amylase, dental/oral examination	S: salivary gland swelling, vomiting<br>O: hyperamylasemia	NR
Kiss *et al.* (1989) (59)	ONCS no follow-up	DSM-III	37/2	24.3	NR	O: upper GI endoscopy	S: vomiting, heartburn, regurgitation<br>O: esophagitis	NR
Koch *et al.* (1998) (60)	OCS no follow-up	DSM-IV	12/0	25	22.5	S: GISS<br>O: GE, electrogastrography	O: GE↑	NR
Metzger *et al.* (1999) (61)	OCS no follow-up	DSM-III-R	17/0	24.5	NR	S: SCQ<br>O: serum & urine amylase, salivary gland biometry	S: vomiting<br>O: salivary gland hypertrophy	NA
Mond *et al.* (2010)* (62)	OCS no follow-up	DSM-IV	5/0 (24/0[1])	NR (29.0[1])	NR (29.2[1])	S: SCQ	S: diarrhea & GI-symptoms not specified	NA
Nickl *et al.* (1996) (63)	OCS no follow-up	DSM-III-R	8/0	26	NR	S: SCQ<br>O: esophageal motor activity & manometry	S: dysphagia, nausea, cramps<br>O: esophagal parameters ↔	NR
Ogawa *et al.* (2004)* (13)	OCS no follow-up	DSM-IV	15/0	23	NR	S: SCQ<br>O: electrogastrography	S: heartburn, epigastric pain, nausea<br>O: gastric electrical dysrhythmia	NR
Ogren *et al.* (1987) (64)	ONCS no follow-up	DSM-III	14/0	23.3	NR	O: Dental/oral examination	S: salivary gland swelling<br>O: dental erosion, caries	NA
Palla & Litt (1988)* (14)	ONCS no follow-up	DSM-III	18/NR	16.8	NR	O: Dental/oral examination	S: salivary gland swelling, heartburn, regurgitation, abdominal fullness, constipation, meteorism<br>O: esophagitis	NR
Paszynska *et al.* (2006) (65)	OCS no follow-up	DSM-IV	33/NR	21.2	NR	O: salivary flow rate	O: salivary flow rate↓	NA
Paszynska *et al.* (2013) (66)	OCS, NRNCT 1-7 m follow-up	DSM-IV ICD-10	33/0	21.2	NR	O: salivary flow rate, salivary electrolytes	S: vomiting<br>O: salivary flow rate↓	Fluoxetine
Riad *et al.* (1991) (67)	OCS no follow-up	DSM-III	28/2	24.6	NR	O: salivary protein measurement, salivary flow rate	S: salivary gland swelling<br>O: salivary amylase↑, salivary flow rate↓	NR
Roberts & Li (1987)* (15)	ONCS no follow-up	NR	30/0	23.5	NR	O: Dental/oral examination	O: dental erosion	NR
Roberts *et al.* (1989) (68)	OCS no follow-up	NR	13/0	26.2	NR	S: SCQ<br>O: salivary amylase, dental/oral examination	S: salivary gland swelling, xerostomia, regurgitation, dysphagia<br>O: absence of gag reflexes, abnormal oropharyngeal swallow patterns	NR
Robertson & Millar (1999) (69)	OCS no follow-up	NR	11/0	NR	22.7	O: serum amylase	S: vomiting<br>O: hyperamylasemia	NA

Supplement Table 1 – Group 1: Human Studies with ED diagnosis, excluding case reports

Author (year)	Study type & follow-up	ED criteria	Characterization n/m	Age (years)	BMI	GI-related measurements S+O	GI outcomes S+O	Intervention
Robinson (1989)* (45)	OCS<br>follow-up after reaching normal weight	NR	10/NR	NR	NR	S: SCQ<br>O: GE	S: nausea, abdominal fullness, decreased satiety<br>O: GE↑	NR
Rothstein (1998) (70)	NRNCT<br>no follow-up	DSM-IV-R	8/0	28.0	NR	S: SCQ<br>O: dental/oral examination, esophagram	S: *pre* heartburn<br>O: *pre* gastroesophageal reflux<br>S+O: *post* improved after treatment in compliant patients	Omeprazol<br>Cisapride
Rytomaa *et al.* (1998) (71)	OCS<br>no follow-up	DSM-III-R	35/0	25.3	NR	S: SCQ<br>O: salivary bacteria, dental/oral examination, dental radiograph	S: xerostomia<br>O: dental erosion, caries, salivary flow rate↓	NR
Salvioli *et al.* (2013)* (47)	NRNCT<br>1 & 6 m follow-up	DSM-IV-R	9/NR<br>(48/7[1])	NR	NR	S: WCS, EGI	S: heartburn, epigastric pain, dysphagia, regurgitation, abdominal pain, nausea, abdominal fullness, fulfilled IBS crit., decreased appetite, abdominal distension	NRP, PT
Satoh & Yoshihara (2004)* (48)	OCS<br>no follow-up	NR	4/NR	NR	NR	O: parotid gland biopsy	O: hyperamylasemia, alteration of parotidal gland cells	NA
Scheutzel & Gerlach (1991)* (17)	OCS<br>no follow-up	DSM-III-R	20/1	24.5	NR	O: salivary & serum amylase, dental/oral examination, salivary flow rate	S: salivary gland swelling<br>O: hyperamylasemia	NR
Sherman *et al.* (1993)* (49)	OCS<br>no follow-up	„according to the estab-lished crit.“	18/0	NR	NR	O: serum Helicobacter pylori IgG antibody	S: vomiting, heartburn, eructation, abdominal pain, GI bleeding, constipation, diarrhea<br>O: Helicobacter pylori infection↔	NR
Shih *et al.* (1987) (72)	OCS, NRNCT<br>regular follow-ups for 3 m	DSM-III	20/0	24.4	NR	S: SCQ<br>O: GE	S: vomiting, constipation, abdominal fullness, epigastric pain, nausea, meteorism<br>O: *pre* GE↑&↓ → *post* MCP improved delayed GE in most patients	MCP
Tylenda *et al.* (1991) (73)	OCS<br>no follow-up	DSM-III<br>Feighner‘s crit.	15/0	25.3	NR	O: salivary flow rate, dental/oral examination	O: salivary flow rate↓	NA
Valena *et al.* (2002) (74)	OCS<br>no follow-up	SCQ, „self-diagnosis“	9/0	32.0	NR	S: SCQ<br>O: dental/oral examination	S: xerostomia, vomiting<br>O: dental erosion	NA
Walsh *et al.* (1990) (75)	RCT<br>follow-up every 1 to 2 w (up to 14x)	DSM-III-R	40/0	NR	21.7	O: serum & salivary amylase	S: vomiting<br>O: salivary amylase↑ → desipramine did not affect serum amylase levels, no difference between medication group and controls	Desipramine
Walsh *et al.* (2003) (76)	OCS, NRNCT<br>48 d follow-up	DSM-IV	16/0	26.7	22.3	S: SCQ<br>O: gastric relaxation, minimal gastric distending pressure	S: vomiting<br>O: *pre* postprandial gastric relaxation↓, minimal gastric distending pressure↔ → *post* p.p. gastric relaxation NR, m.g.d. pressure increased	PT

Supplement Table 1 – Group 1: Human Studies with ED diagnosis, excluding case reports

Author (year)	Study type & follow-up	ED criteria	Characterization			GI-related measurements S+O	GI outcomes S+O	Intervention
			n/m	Age (years)	BMI			
Winstead & Willard (2006)* (21)	OCS 1 y follow-up	SCQ	11/NR (63/6[1])	NR (27.6[1])	NR	S: SCQ	S: heartburn	NA
Wockel *et al.* (2007)* (52)	OCS no follow-up	NR	9/0	17.1	22.6	O: papilla counting, taste strips	O: amount of papillas on tongue↓	NA
Wolff *et al.* (1968)* (23)	ONCS	NR	3/0	NR (35.1[1])	15.6	S: SCQ	S: vomiting, nausea, constipation	NA
Zimmerli *et al.* (2006) (77)	OCS no follow-up	DSM-IV	16/0	27.9	22.3	O: gastric capacity	S: vomiting<br>O: gastric compliance↔	NA
Studies with Binge-eating Disorder								
Cremonini *et al.* (2009) (78)	OCS no follow-up	Modified Q-EWP-R	111/NR	45.1	31.1	S: SCQ, BDQ	S: heartburn, epigastric pain, dysphagia, regurgitation, abdominal pain, constipation, meteorism, diarrhea, urgency, anal blockage	NA
Crowell *et al.* (1994) (79)	OCS no follow-up	DSM-IV	87/0	45.1	NR	S: BSQ	S: vomiting, eructation, abdominal pain, nausea, fulfilled IBS crit., indigestion, constipation, meteorism, diarrhea	NA
Geliebter *et al.* (2004) (80)	OCS no follow-up	Q-EWP	11/0	29.0	36.6	O: gastric capacity	O: stomach capacity↑	NA
Gowen *et al.* (1999)* (81)	OCS no follow-up	NR	16/8	NR	NR	O: upper GI endoscopy	O: esophagitis, gastroesophageal reflux, ulcer, SMA syndrome, intussusception	NA
Kinzl *et al.* (1993)* (41)	OCS no follow-up	DSM-III-R	NR	NR	NR	O: salivary & serum amylase, dental/oral examination	S: salivary gland swelling, vomiting<br>O: hyperamylasemia	NR
Levy *et al.* (2005) (82)	OLCS 24 mo follow-up	Q-EWP	56/NR	NR	NR	S: ROME II	S: abdominal pain, constipation, meteorism	Telephone- & mail-based interventions for weightloss
Mond *et al.* (2010)* (62)	OCS no follow-up	DSM-IV	6/0	NR (29.0[1])	NR (29.2[1])	S: SCQ	S: diarrhea, GI symptoms not specified	NA
Peat *et al.* (2013) (83)	OCS no follow-up	DSM-IV	403/41	NR	26.9	S: SCQ	S: heartburn, fulfilled IBS crit., constipation, meteorism, diarrhea	NA
Thornton (Watson) *et al.* (2017) (84)	OCS no follow-up	DSM-IV	850/39	NR	NR	S: SCQ	S: GI symptoms not specified	NA
Thornton (Trace) *et al.* (2017) (85)	OCS no follow-up	DSM-IV	22/0	31.7	NR	S: SCQ	S: GI symptoms not specified	NA
Exact eating disorder not reported								
Lobera *et al.* (2011) (86)	OCS no follow-up	DSM-IV-R	78/8	22.9	NR	S: SCQ, ROME III	S: epigastric pain, eructation, abdominal pain, nausea, decreased satiety, meteorism	NA

* also assessing at least one other ED and therefore presented several times in the tables
[1] for all EDs considered in this study
[2] for both AN subtypes considered in this study
[3] Weight and /or % of ideal bodyweight were reported in brackets if BMI was not reported
[4] % of ideal bodyweight was reported in brackets if BMI and weight were not reported
Time: d=day(s), w=week(s), m=month(s), y=year(s)
Study types: NRCT=Non-randomized controlled trial, NRNCT=Non-randomized non-controlled trial, OCS=Observational controlled tudy, OLCS=Observational longitudinal controlled study, OLS=Observational longitudinal study, ONCS=Observational non-controlled study, RCT=Randomized controlled trail, RNCT=Randomized non-controlled trial
ED criteria: DSM=Diagnostic and Statistical Manual of Mental Disorders, R=Revised, APA=American Psychiatric Association, AMA=American Medical Association, crit.=criteria
Questionnaires: AOD-S=Altomare's obstructed defecation score, BDQ=Bowel Disease Questionnaire, BSQ=Bowel Symptom Questionnaire, CSI=Children's Somatization Inventory, EGI=Questionnaire on esophago-gastrointestinal symptoms, FISI=Faecal Incontinence Severity Index, G-Q=Gastro-Questionnaire, GCSI=Gastroparesis Cardinal Symptom Index, GIM=Gastrointestinal Impairment Score, GIS=Gastrointestinal Symptom Score, GISS=GI Symptom Survey, IBS-Q=Irritable Bowe Syndrome Questionnaire, LDQ=Leeds Dyspepsia Questionnaire, MMPI=Minnesota Multiphasic Personality Inventory, Q-EWP=Questionnaire on Eating and Weight Patterns, ROME=Rome Diagnostic Questionnaire for FGIDs, SCL-90=Symptom Check List-90, SCQ=Self-constructed questionnaire (including also anamnesis/medical history), WCS=Wexner Constipation Score
AN=Anorexia nervosa (b/p=binge/purge subtype, r=restrictive subtype), ARFID=Avoidant/Restrictive Food Intake Disorder, BED=Binge-eating Disorder, BMI=body mass index, BN=Bulimia nervosa, BW=body weight, CT=computed tomography, FGID=Functional Gastrointestinal Disorder, GE=gastric emtying time, GI=gastrointestinal, IBS=Irritable Bowel Syndrome, IBW=ideal body weight, LES=lower esophagal sphincter, LF=Liquid formula, m=males, M=Medication, med.=median, MRI=magnetic resonance imaging, n=sample size, NIT=Non-invasive treatment, NA=Not applicable, NR=Not reported, NRP=Nutritional Rehabilitation program, O=objective, OSFED=Other SMA=Superior mesenteric artery syndrome, Specified Feeding and Eating Disorder, SSRI=Selective serotonine reuptake inhibitors, PT=Psychotherapy, S=subjective, UES=upper esophagal sphincter, UFED=Unspecified Feeding and Eating Disorder

Supplement Table 2 – Group 2: Case reports with ED diagnosis

**Author (year)**	**Characterization** n/m	Age (years)	BMI (weight/%IBW)[3]	**GI-related measurements S+O**	**GI outcomes S+O**	**Intervention**	**Lethal outcome**
Anorexia nervosa, restrictive subtype							
Brook (1977) (87)	1/0	17	NR (35%)	PE, X-ray	S: abdominal pain, abdominal distension, nausea<br>O: gastric dilatation	NRP<br>M: chlorpromazine, MAO inhibitors	-
Buchman *et al.* (1994) (88)	1/0	21	14.3	X-ray, esophageal & gastroduodenal manometry,<br>small bowel transit time, orocecal transit time, esophageal pHmetry	S: abdominal pain, nausea<br>O: duodenal dilatation and dysmotility	NRP, parenteral nutrition<br>NIT: nasogastric drainage	-
El Ghoch *et al.* (2016) (89)	1/0	26	13.2	Stool examination, lower GI endoscopy	S: abdominal pain, constipation, meteorism<br>O: rectocele, GI bleeding	NRP	-
Elizondo-Romo *et al.* (2018) (90)	1/0	34	6.0	CT	S: abdominal pain<br>O: intestinal pneumatosis	NRP, parenteral nutrition<br>NIT: nasogastric drainage	-
Evans (1968) (91)	1/0	20	NR (39kg)	PE, X-ray	S: vomiting, abdominal pain<br>O: gastric dilatation, wall ischemia and perforation, absence of bowel sounds, signs of peritonitis, peritonitis	M: chlorpromazine, insulin<br>OP: laparotomy, gastrectomy	-
Fenley *et al.* (1990) (92)	1/0	75	14.0	PE	S: constipation<br>O: hyperamylasemia, xerostomia, furrowed tongue,<br>esophagitis, gastric ulcer,<br>colonic perforation, colonic transit time↑, anorectal pressure↑,<br>gastroenteritis, disordered GI motility	NRP, PT	-
Fernandez Lopez *et al.* (2011) (93)	1/0	31	16.7	PE, stool examination, upper GI endoscopy	S: vomiting<br>O: duodenal dilatation, SMA syndrome	M: prokinetics<br>NIT: nasogastric drainage<br>OP: laparotomy, duodenojejunostomy	-
Garcia et al. (2006) (94)	1/1	21	13.3	PE, X-ray, CT	O: intestinalis pneumatosis	NR	-
Goldstein *et al.* (2008) (95)	1/1	19	14.1	X-ray, CT	S: abdominal pain, abdominal fullness, nausea<br>O: gastric dilatation, duodenal dilatation	Parenteral nutrition<br>NIT: nasogastric drainage	-
Gwee *et al.* (2010) (96)	1/0	17	16.4	PE, X-ray, CT	S: vomiting, abdominal pain, abdominal fullness<br>O: gastric dilatation, SMA syndrome	NIT: nasogastric drainage	-
Holmes *et al.* (2012)* (97)	2/0	24<br>25	9.3<br>11.3	PE, X-ray	S: dysphagia<br>O: GER	NR	-
Masuda *et al.* (2001) (98)	1/0	17	14.3	PE, X-ray	S: epigastric pain, abdominal pain, nausea, right iliac fossa pain, decreased appetite, constipation, meteorism<br>O: ileus	NRP, liquid diet, parenteral nutrition	-
Osegueda de Rodriguez *et al.* (2017) (99)	2/0	21<br>17	16.8<br>8.3	CT	S: vomiting, nausea, epigastric pain, abdominal distension<br>O: SMA syndrome, duodenal compression	NRP, nasogastric tube, enteral nutrition, parenteral nutrition<br>OP: laparotomy, gastectomy	-

Supplement Table 2 – Group 2: Case reports with ED diagnosis

Author (year)	Characterization n/m	Age (years)	BMI (weight/%IBW)[3]	GI-related measurements S+O	GI outcomes S+O	Intervention	Lethal outcome
Saito *et al.* (1999) (100)	3/0	18<br>16<br>14	10.1<br>10.4<br>11.1	Bone marrow biopsy	S: gingival bleeding<br>O: GI bleeding	NRP, parenteral nutrition<br>NIT: platelet transfusion	-
Saito *et al.* (2015) (101)	1/0	28	9.3	X-ray, CT, upper GI endoscopy	S: abdominal pain, nausea<br>O: tracheoesophageal fistula, bowel perforation, peritonitis	Enteral nutrition<br>OP: laparotomy, gastrotomy	-
Sansone & Sansone (2003) (102)	1/0	19	11.0	-	S: self-report GE↑	NRP<br>M: metoclopramide	-
Anorexia nervosa, binge/purge subtype							
*Abdu et al. (1987)** (103)	1/0	14	14.3	PE, X-ray	S: abdominal pain, abdominal distension<br>O: gastric dilatation, wall ischemia, necrosis & perforation, duodenal dilatation, colonic dilatation,<br>absence of bowel sounds	NRP, enteral & parenteral nutrition<br>NIT: nasogastric drainage, gastric decompression, resuscitation<br>OP: laparotomy, gastrotomy, gastrectomy, esophagojejunostomy, appendectomy	-
Adson *et al.* (1997)* (104)	1/0	30	NR (31.8kg)	X-ray, CT	S: vomiting, inability to vomit, abdominal distension, right iliac fossa pain<br>O: gastric dilatation	NIT: nasogastric drainage<br>OP: laparotomy	-
Albano *et al.* (2017) (105)	1/0	19	NR	X-ray	S: vomiting, epigastric pain, nausea, decreased appetite<br>O: gastric dilatation, GE↑,<br>SMA syndrome	NRP	-
Berke & Calcaterra (1985) (106)	1/0	49	NR	PE, X-ray, CT, oral biopsy	S: parotidal gland swelling, vomiting<br>O: parotidal gland swelling	OP: operation, salivary gland resection	-
Birmingham *et al.* (2007) (107)	1/0	19	NR	PE, CT, upper GI endoscopy	S: vomiting, hematemesis, GE↑, bezoar in vomit, nausea, constipation, diarrhea<br>O: parotidal gland swelling, hiatus hernia	NR	-
Browning (1977) (108)	2/0	16<br>19	NR (30.4kg)<br>NR (31.3kg)	Post-mortem autopsy	S: vomiting, abdominal pain, abdominal distension, abdominal fullness<br>O: tracheoesophageal fistula, gastric dilatation, necrosis and perforation	Enteral nutrition, liquid diet, PT<br>NIT: resuscitation,<br>OP: laparotomy, gastrectomy	+<br>-
Bruno *et al.* (2015)* (109)	2/0	25<br>24	NR<br>18.5	PE, dental/oral examination,<br>lower GI endoscopy, anorectal manometry, colonic transit time	S: regurgitation, constipation<br>O: dental erosion, delayed colonic transit, increased anorectal pressure	M: prokinetics	-
Buchanan & Fortune (1994) (110)	1/1	25	NR (52.0kg)	Dental/oral examination, X-ray, oral biopsy	S: parotidal gland swelling, xerostomia, vomiting, nausea<br>O: submandibular gland hypertrophy, dental erosion	NRP, PT	-
De Caprio *et al.*<br>(2000) (111)	1/1	16	13.3	PE, X-ray, CT, upper GI endoscopy	S: vomiting, epigastric pain, abdominal distension<br>O: gastric dilatation, gastritis,<br>pyloric stenosis	Parenteral nutrition<br>NIT: nasogastric drainage	-

Supplement Table 2 – Group 2: Case reports with ED diagnosis

Author (year)	Characterization			GI-related measurements S+O	GI outcomes S+O	Intervention	Lethal outcome
	n/m	Age (years)	BMI (weight/%IBW)[3]				
Di Luca et al. (2019) (112)	1/0	18	16.0	PE, CT, post-mortem autopsy	S: abdominal pain, abdominal fullness, abdominal distension<br>O: gastric dilatation, absence of bowel sounds, faecal incontinence	Resuscitation	+
Dumouchel *et al.* (2017) (113)	1/0	26	14.1	PE, CT	S: abdominal pain<br>O: gastric dilatation	M: metoclopramide, analgesics<br>NIT: nasogastric drainage	-
Eraslan *et al.* (2009) (114)	1/0	25	12.1	-	S: vomiting<br>O: GER	NRP, PT, M: fluoxetine, amitryptiline, olanzapine, omeprazole<br>OP: laparotomy, operation, Nissen fundoplicatio	-
Franco-Lopez *et al.* (2012) (115)	1/0	31	16.6	PE, X-ray, CT	S: vomiting, inability to vomit, abdominal pain, abdominal distension<br>O: gastric dilatation	PT, NIT: nasogastric drainage,<br>OP: laparotomy, gastrotomy	-
Gore & Port (1982) (116)	1/0	13	NR	PE, X-ray	O: gastritis	NR	-
Hollatz & Ziolko (1976) (117)	2/0	22<br>44	12.9<br>15	X-ray, post-mortem autopsy	O: ileus, gastroenterocolitis	Parenteral nutrition	+<br>+
Holmes *et al.* (2012)* (97)	1/0	33	12.3	PE, X-ray	Upper GI: dysphagia<br>O: GER	NR	-
Imai & Michizawa (2013) (118)	1/0	26	15.6	Dental/oral examination, oral biopsy	S: ulcerative stomatitis, vomiting<br>O: submandibular gland hypertrophy, necrotizing sialometaplasia, dental erosion	NR	-
Kim *et al.* (2009) (119)	1/0	34	NR	PE, ultrasonograpy, CT, upper GI endoscopy	S: abdominal pain<br>O: esophageal dilatation, gastric dilatation, gastric ulcera, duodenal dilatation	NIT: nasogastric drainage, resuscitation	-
Kim *et al.* (2011) (120)	1/0	26	16.2	PE, X-ray, CT, upper GI endoscopy	S: abdominal pain<br>O: gastric dilatation, duodenal compression, absence of bowel sounds	NRP,<br>NIT: nasogastric drainage, PT	-
Koyazounda *et al.* (1985) (121)	1/1	34	NR	X-ray	S: diarrhea<br>O: gastric dilatation, wall ischemia and necrosis	NIT: resuscitation<br>OP: laparotomy, gastrectomy	-
Lin *et al.* (1995) (122)	1/1	58	NR	Ultrasonography, stool examination, X-ray, upper & lower GI endoscopy	S: abdominal distension<br>O: gastric dilatation, gastric ulcera, gastritis, intestinalis pneumatosis	NIT: nasogastric drainage<br>OP: laparotomy, gastrectomy, esophagojejunostomy	-
Mascolo *et al.* (2015) (123)	1/0	47	10.7	PE, CT	S: vomiting, abdominal pain, nausea<br>O: gastric dilatation, SMA syndrome	NRP, liquid diet	-
Mathevon *et al.* (2004) (124)	1/0	25	15.4	Upper GI endoscopy	S: vomiting, abdominal pain<br>O: gastric dilatation, gastritis, absence of bowel sounds	NRP,<br>NIT: nasogastric tube	-
Matsuyama *et al.* (2008) (125)	1/0	24	13.3	X-ray	S: abdominal distension<br>O: gastric dilatation	NIT: nasogastric drainage	-
Mehler & Weiner (2007) (126)	2/0	24<br>31	NR<br>NR	PE	S: vomiting, abdominal pain<br>O: SMA syndrome, cathartic colon	Parenteral nutrition<br>NIT: nasogastric drainage<br>OP: colectomy	-

Supplement Table 2 – Group 2: Case reports with ED diagnosis

Author (year)	Characterization			GI-related measurements S+O	GI outcomes S+O	Intervention	Lethal outcome
	n/m	Age (years)	BMI (weight/%IBW)[3]				
Mignogna *et al.* (2004) (127)	1/0	28	NR	Dental/oral examination, CT	S: parotidal gland swelling<br>O: parotidal gland hypertrophy, minor salivary gland hypertrophy	Mt: pilocarpine hydrochloride	-
Mitchell & Norris (2013) (128)	1/0	16	18.5	PE	S: constipation, rectal prolapse	Increased fluid and fiber therapy<br>NIT: physiotherapy	-
Pacciardi *et al.* (2015) (129)	1/0	37	13.5	PE, upper GI endoscopy	S: heartburn<br>O: Barrett's esophagus	NRP, PT<br>M: SSRIs, benzodiazepines, metoclopramide	-
Pandey *et al.* (2009) (130)	1/0	32	15.0	PE, CT	S: abdominal pain, nausea<br>O: parotidal gland hypertrophy, gastric dilatation	NIT: nasogastric drainage	-
Repesse *et al.* (2013) (131)	1/0	18	11.4	PE, CT	S: abdominal pain<br>O: gastric dilatation	NIT: nasogastric drainage	-
Roggo & Filippini (1989) (132)	1/0	25	NR	X-ray	S: dysphagia, retrosternal pain<br>O: esophageal perforation	DT: parenteral nutrition<br>M: antibiotics	-
Roseborough & Felix (1994) (133)	1/0	43	NR	X-ray, lower GI endoscopy	O: gastric dilatation and perforation, acute abdomen	NIT: resuscitation<br>OP: laparotomy	+
Sastre *et al.* (2015) (134)	1/0	23	NR	PE, CT, upper GI endoscopy	S: inability to vomit, abdominal pain, nausea<br>O: gastric dilatation, wall ischemia, necrosis & perforation, bowel necrosis	OP: laparotomy, gastrectomy	+
Schechter *et al.*(1986) (135)	1/0	20	NR (38.5kg)	PE, X-ray	S: inability to vomit, epigastric pain, abdominal pain, abdominal distension<br>O: esophageal perforation, gastric dilatation, absence of bowel sounds	M: metoclopramide<br>NIT: nasogastric drainage	-
Sinicina *et al.* (2005) (136)	1/0	19	17.9	Post-mortem autopsy	O: gastric dilatation and perforation	NR	+
Trott *et al.* (1990) (137)	1/0	17	11.8	PE, X-ray	O: gastric dilatation and perforation, duodenal compression, SMA syndrome, acute abdomen	OP: laparotomy	-
Tweed-Kent *et al.* (2010) (138)	1/0	26	18.7	PE, X-ray, CT	S: inability to vomit, epigastric pain, abdominal pain, nausea<br>O: gastric dilatation, necrosis, wall ischemia	NIT: nasogastric drainage, resuscitation<br>OP: laparotomy, gastrotomy	-
van Dijk *et al.* (1994) (139)	1/0	31	13.6	PE, X-ray	S: abdominal pain, abdominal distension<br>O: gastric dilatation and perforation, signs of peritonitis	NIT: nasogastric drainage<br>OP: laparotomy, gastrectomy	+
van Eetvelde *et al.* (2014) (140)	1/0	19	NR	CT, upper GI endoscopy	S: inability to vomit, dysphagia<br>O: gastric dilatation and wall ischemia, acute abdomen	NIT: resuscitation<br>OP: laparoscopy, gastrectomy	-
Walsh *et al.* (1981) (141)	3/0	23<br>29<br>29	15.0<br>NR (30.6kg)<br>14.0	PE, oral biopsy	S: parotidal gland swelling<br>O: parotidal, submandibular and minor salivary gland hypertrophy	M: amitryptiline, tryptophan<br>PT	-
Watanabe *et al.* (2008) (142)	1/0	31	16.2	Post-mortem autopsy	O: gastric dilatation and necrosis, bloody ascites, GI bleeding	NR	+

Supplement Table 2 – Group 2: Case reports with ED diagnosis

Author (year)	Characterization			GI-related measurements S+O	GI outcomes S+O	Intervention	Lethal outcome
	n/m	Age (years)	BMI (weight/%IBW)[3]				
Willeke *et al.* (1996) (143)	1/0	17	14.7	PE, ultrasonography, X-ray, upper GI endoscopy	S: inability to vomit, abdominal pain, abdominal fullness<br>O: gastric necrosis and perforation, peritonitis	M: katecholamines, antibiotics<br>NIT: nasogastric drainage<br>OP: laparotomy, gastrectomy	-
Yamada *et al.* (2001) (144)	1/0	41	12.2	PE, X-ray, lower GI endoscopy,<br>post-mortem autopsy	S: vomiting, epigastric pain, abdominal distension, nausea, constipation<br>O: duodenal dilatation, ileus, colonic dilatation and perforation, absence of bowel sounds, GI bleeding, bloody ascites, bowel necrosis & perforation, peritonitis	NIT: nasogastric drainage<br>OP: laparotomy	+
Yamaguchi *et al.* (1992) (145)	1/0	16	12.7	PE, upper GI endoscopy	S: vomiting, epigastric pain<br>Lower GI: diarrhea	supplement: zinc	-
Anorexia nervosa, subtype not clear							
De Silva *et al.* (1998) (146)	1/0	28	NR	PW, dental/oral examination	S: vomiting<br>O: parotidal gland swelling, gastric dilatation, duodenal dilatation, SMA syndrome	M: prokinetics<br>OP: laparotomy	-
Diamanti *et al.* (2011) (147)	1/0	17	10.0	PE, X-ray, stool examination	S: vomiting, abdominal pain, abdominal distension, diarrhea<br>O: gastric dilatatio, GI bleeding, intestinalis pneumatosis	Parenteral nutrition<br>NIT: nasogastric drainage	-
Dreznik *et al.* (2001) (148)	3/0	20<br>18<br>24	NR (45.0kg)<br>NR<br>NR (42.0kg)	PE, lower GI endoscopy	S: constipation, rectal prolapse	OP: laparotomy, proctosigmoidectomy	-
Dzirlo *et al.* (2013) (149)	1/0	35	15.3	PE, stool examination, ultrasonography, CT	S: diarrhea, meteorism<br>O: intestinalis pneumatosis, ascites	OP: laparoscopy	-
Elbadawy (1992) (150)	1/0	18	12.7	X-ray, upper GI endoscopy	S: vomiting, epigastric pain, heartburn, decreased appetite<br>O: gastric dilatation, SMA syndrome	OP: laparotomy, gastrojejunostomy	-
Kalouche *et al.* (1991) (151)	1/0	20	16.7	PE, X-ray	S: vomiting, abdominal pain, right iliac fossa pain<br>O: gastric dilatation, duodenal dilatation & compression, SMA syndrome	OP: laparotomy	-
Kaye *et al.* (1985) (152)	1/0	17	NR	PE, X-ray	S: vomiting, abdominal pain, nausea, abdominal fullnes, constipation<br>O: colonic dilatation, necrotizing colitis, signs of peritonitis, bowel perforation, peritonitis	NIT: resuscitation<br>OP: laparotomy, colectomy	+
Mearelli *et al.* (2014) (153)	1/1	47	NR	PE, CT, upper GI endoscopy	S: vomiting, abdominal pain<br>O: gastric dilatation, duodenal dilatation & compression, SMA syndrome	Parenteral nutrition<br>NIT: nasogastric drainage<br>OP: operation, Treitz ligament mobilisation	-
Miller *et al.* (1991) (154)	1/0	30	12.2	X-ray, CT	S: abdominal pain, abdominal distension<br>O: intestinalis pneumatosis, volvulus, bowel necrosis	NR	+
Pua (2011) (155)	1/0	27	NR	PE, X-ray, CT	S: abdominal pain<br>O: intussusception	Enteral nutrition	-

Supplement Table 2 – Group 2: Case reports with ED diagnosis

Author (year)	Characterization			GI-related measurements S+O	GI outcomes S+O	Intervention	Lethal outcome
	n/m	Age (years)	BMI (weight/%IBW)[3]				
Sakka *et al.* (1994) (156)	1/0	20	NR (30.0kg)	X-ray	S: abdominal pain, abdominal distension, right iliac fossa pain, diarrhea<br>O: necrotizing colitis	NIT: resuscitation<br>OP: laparotomy, colectomy	+
Vannatta *et al.* (1976) (157)	1/0	17	NR	PE, X-ray	S: vomiting<br>O: duodenal compression, SMA syndrome	OP: laparotomy, gastrojejunostomy, duodenojejunostomy	-
Wu & Guan (2016) (158)	1/0	25	NR	PE, X-ray, CT	S: vomiting, epigastric pain, abdominal distension, constipation<br>O: duodenal compression, intestinalis pneumatosis	Parenteral nutrition<br>NIT: nasogastric drainage	-
Yao *et al.* (2015) (159)	1/0	17	14.0	Ultrasonography, X-ray, CT	S: vomiting, abdominal distension<br>O: gastric dilatation, SMA syndrome, duodenal dilatation and compression	OP: laparoscopy, Treitz ligament mobilisation, gastrojejunostomy, duodenojejunostomy	-
Anorexia nervosa, binge/purge subtype or Bulimia nervosa (not clear)							
Antic *et al.* (2014) (160)	1/0	19	NR	CT, upper GI endoscopy	S: inability to vomit, abdominal pain, abdominal distension<br>O: gastric dilatation and necrosis, ascites	NIT: nasogastric drainage	-
Beiles et al. (1992) (161)	1/0	24	NR	X-ray	S: vomiting, abdominal pain<br>O: gastric dilatation, necrosis and perforation, absence of bowel sounds	OP: laparotomy	-
Carlson (2009) (162)	1/0	32	NR	PE, oral biopsy	S: painful intraoral salivary gland swelling<br>O: necrotizing sialometaplasia, minor salivary gland hypertrophy	OP: operation, salivary gland resection	-
Delap *et al.* (1996) (163)	1/0	22	NR	PE, X-ray, CT	S: throat soreness<br>O: retropharyngeal abscess	NRP,<br>NIT: nasogastric drainage<br>OP: operation, Treitz ligament mobilisation	-
Devitt & Stamp (1983) (164)	1/0	23	NR	X-ray, post-mortem autopsy	S: inability to vomit, abdominal pain, abdominal distension<br>O: gastric dilatation, wall ischemia and necrosis, GI bleeding, bowel necrosis	NIT: nasogastric drainage<br>OP: laparotomy	+
Maung *et al.* (2017) (165)	1/0	26	NR	CT, upper GI endoscopy	S: inability to vomit, abdominal pain, abdominal distension<br>O: gastric dilatation, gastric wall ischemia, duodenal decompression	NIT: nasogastric drainage	-
Navab *et al.* (1996) (166)	1/1	37	NR	PE, dental/oral examination, CT, upper GI endoscopy	S: vomiting, heartburn, hematemesis, regurgitation<br>O: GER, esophagitis, Barrett's esophagus, esophageal candidosis, esophageal adenocarcinoma, hiatus hernia	M: fluconazole<br>OP: laparotomy, gastrectomy	-
Patocskai & Thomas (2002) (167)	1/0	28	NR	PE, X-ray	S: inability to vomit, abdominal pain, abdominal distension<br>O: gastric dilatation and necrosis, signs of peritonitis	OP: laparotomy, gsatrectomy, esophagoantral anastomosis	-
Petrin *et al.* (1990) (168)	1/0	25	NR (42.0kg)	PE, X-ray	S: vomiting, abdominal pain<br>O: gastric dilatation, wall ischemia, necrosis and perforation	Enteral nutrition<br>NIT: nasogastric drainage<br>OP: laparotomy, gastrectomy, esophagojejunostomy	-

Supplement Table 2 – Group 2: Case reports with ED diagnosis

Author (year)	Characterization n/m	Age (years)	BMI (weight/%IBW)[3]	GI-related measurements S+O	GI outcomes S+O	Intervention	Lethal outcome
Reichel *et al.* (2003) (169)	1/0	28	NR	PE, ultrasonography, MRI, oral biopsy	S: parotidal gland swelling	PT,M: pilocarpin<br>OP: operation, salivary gland resection	-
Rosset & Yuen (2015) (170)	1/0	41	NR	PE, CT, upper GI endoscopy	S: abdominal pain, abdominal distension<br>O: gastric dilatation and wall ischemia, intestinalis pneumatosis	NIT: nasogastric drainage, resuscitation<br>OP: laparotomy, gastrectomy, esophagojejunostomy	-
Bulimia nervosa							
Abdu *et al.* (1987)* (103)	1/0	17	NR	PE, X-ray	S: abdominal pain, abdominal distension<br>O: gastric dilatation, wall ischemia, necrosis and perforation, duodenal dilatation, colonic dilatation, absence of bowel sounds	NRP, enteral & parenteral nutrition<br>NIT: nasogastric drainage, gastric decompression, resuscitation<br>OP: laparotomy, gastrotomy, gastrectomy, esophagojejunostomy, appendectomy	-
Adson *et al.* (1997)* (104)	1/0	35	20.5	X-ray, CT	S: vomiting, inability to vomit, abdominal distension, right iliac fossa pain<br>O: gastric dilatation	NIT: nasogastric drainage<br>OP: laparotomy	-
Bravender & Story (2007) (171)	1/0	21	20.9	PE, X-ray, CT	S: inability to vomit, abdominal pain, abdominal distension, nausea<br>O: gastric dilatation, absence of bowel sounds	NIT: nasogastric drainage	-
Bruno *et al.* (2015)* (109)	1/1	40	NR	PE, dental/oral examination, lower GI endoscopy, anorectal manometry, colonic transit time	S: regurgitation, constipation<br>O: dental erosion, delayed colonic transit, increased anorectal pressure	M: prokinetics	-
Cosins *et al.* (1986) (172)	1/0	20	18.6	X-ray	S: parotidal gland swelling<br>O: parotidal gland hypertrophy	M: „Ethyblok“	-
Cuellar *et al.* (1988) (173)	5/1	22<br>24<br>34<br>20<br>34	19.8<br>NC<br>22<br>24.5<br>20.5	PE, upper GI endoscopy, esophageal manometry, esophageal transit time, GE	S: vomiting, epigastric pain, heartburn, dysphagia, hematemesis, abdominal pain, nausea, decreased appetite<br>O: GER, esophagitis, gastric ulcer, gastritis, GE↑, duodenal ulcer, GI bleeding	alcohol abstinence, PT<br>M: antidepressants, histamine receptor antagonists<br>NIT: nasogastric drainage	-
Czarnecki & O‘Coclain (2002) (174)	1/0	17	NR	PE, CT	S: inability to vomit, abdominal pain, abdominal distension<br>O: gastric dilatation	PT, NIT: nasogastric drainage	-
Elsharif *et al.* (2014) (175)	1/0	18	NR	CT	S: abdominal pain, abdominal distension<br>O: gastric dilatation and necrosis, bowel necrosis	DT: parenteral nutrition<br>NIT: resuscitation<br>OP: laparotomy, gastrotomy, gastrectomy, esophagojejunostomy, colectomy	-
Garcia Vasquez *et al.* (2014) (176)	1/0	31	NR	CT	S: vomiting, abdominal distension<br>O: gastric dilatation, duodenal dilatation	NIT: nasogastric drainage	-

Supplement Table 2 – Group 2: Case reports with ED diagnosis

Author (year)	Characterization n/m	Age (years)	BMI (weight/%IBW)[3]	GI-related measurements S+O	GI outcomes S+O	Intervention	Lethal outcome
Herrlinger & Gundlach (2001) (177)	1/0	22	20.1	PE, dental/oral examination, oral biopsy, serum amylase	S: parotidal gland swelling, painful intraoral salivary gland swelling<br>O: parotidal and submandibular gland hypertrophy, hyperamylasemia	NR	-
Hohenauer & Dunser (2011) (178)	1/0	21	NR	Upper GI endoscopy	O: gastric dilatation, gastric wall ischemia	NIT: gastric decompression	-
Jones & Morgan (2012) (179)	1/1	NR	NR	Upper GI endoscopy	S: vomiting, eructation, abdominal pain, meteorism	PT	-
Kashyap *et al.* (2009) (180)	1/0	36	NR	PE, ultrasonography, X-ray, CT	S: vomiting, abdominal pain, nausea, diarrhea<br>O: gastric dilatation	NIT: nasogastric drainage<br>OP: laparotomy, gastrotomy	-
Kim *et al.* (2017) (181)	1/0	24	NR	CT, upper GI endoscopy	S: vomiting, abdominal pain, abdominal distension, nausea<br>O: gastric dilatation, gastric atony, SMA syndrome absence of bowel sounds, GI bleeding	M: digestives, enema<br>NIT: nasogastric drainage, resuscitation<br>OP: laparotomy, gastrotomy	+
Kimura *et al.* (2015) (182)	1/0	34	21.1	PE, X-ray, CT, upper GI endoscopy	S: inability to vomit, abdominal distension<br>O: gastric dilatation, pyloric stenosis	NIT: nasogastric drainage, resuscitation<br>OP: laparotomy, gastroejunostomy	-
Lambeck & Hacki (1997) (183)	1/0	29	NR	-	S: vomiting, heartburn, eructation	PT	-
Levin *et al.* (1980) (184)	7/0	19<br>32<br>21<br>21<br>23<br>15<br>24	19.7<br>18.8<br>NR (103%)<br>NR (98%)<br>NR (100%)<br>NR (111%)<br>NR (111%)	PE, ultrasonography, oral biopsy	S: parotidal gland swelling<br>Upper GI: vomiting<br>O: parotidal and submandibular gland hypertrophy	NR	-
Malik *et al.* (1997) (185)	7/0	29	NR	PE, lower GI endoscopy	S: vomiting, constipation, diarrhea, rectal prolapse, rectal bleeding<br>O: rectal polyp	OP: laparotomy, sigmoid resection	1 of 7
Mitchell *et al.* (1982) (186)	1/0	21	NR	PE	S: vomiting, inability to vomit, abdominal pain<br>O: gastric dilatation	NIT: nasogastric drainage	-
Park *et al.* (2009) (187)	1/0	36	NR	CT	S: parotidal gland swelling<br>O: parotidal and minor salivary gland hypertrophy	Mt: pilocarpine hydrochloride	-
Pedrolli *et al.* (2015) (188)	1/0	48	19.3	PE, X-ray, ultrasonography, upper GI endoscopy	S: vomiting, epigastric pain, heartburn, nausea<br>O: acute abdomen	NR	-
Sansone *et al.* (2005) (189)	1/0	23	NR	PE	S: vomiting, hematemesis<br>O: gastritis, gastric ulcera	PT, M: sertraline, naproxen	-
Schoning *et al.* (1998) (190)	2/0	20<br>21	NR<br>NR	Dental/oral examination, X-ray, oral biopsy	S: vomiting<br>O: necrotizing sialometaplasia, minor salivary gland hypertrophy, oral ulcera	NR	-

Supplement Table 2 – Group 2: Case reports with ED diagnosis

**Author (year)**	**Characterization** n/m	Age (years)	BMI (weight/%IBW)[3]	**GI-related measurements S+O**	**GI outcomes S+O**	**Intervention**	**Lethal outcome**
Taylor & Sneddon (1987)(191)	1/0	26	NR	Dental/oral examination, X-ray, CT	S: parotidal gland swelling<br>O: parotidal gland hypertrophy	NR	-
Usui *et al.* (2016) (192)	1/0	NR	18.8	X-ray, CT	S: vomiting, abdominal pain, abdominal distension<br>O: gastric dilatation	NR	+
Vavrina *et al.* (1994) (193)	1/1	24	19.8	Ultrasonography, CT, serum amylase, oral biopsy	S: parotidal gland swelling, vomiting<br>O: parotidal and submandibular gland hypertrophy, amylasemia	PT	-
Wilson & Price (2003) (194)	1/0	26	NR	PE	S: parotidal gland swelling<br>O: parotidal and submandibular gland hypertrophy, parotid fistula	PT<br>OP: operation, salivary gland resection	-
Winstead & Willard (1983) (195)	1/0	24	18.6	PE	S: vomiting, heartburn, abdominal pain	NRP, PT	-

* also assessing at least one other ED and therefore presented several times in the tables
[1] for all EDs considered in this study
[2] for both AN subtypes considered in this study
[3] Weight and /or % of ideal bodyweight were reported in brackets if BMI was not reported
[4] % of ideal bodyweight was reported in brackets if BMI and weight were not reported
Time: d=day(s), w=week(s), m=month(s), y=year(s)
Study types: NRCT=Non-randomized controlled trial, NRNCT=Non-randomized non-controlled trial, OCS=Observational controlled tudy, OLCS=Observational longitudinal controlled study, OLS=Observational longitudinal study, ONCS=Observational non-controlled study, RCT=Randomized controlled trail, RNCT=Randomized non-controlled trial
ED criteria: DSM=Diagnostic and Statistical Manual of Mental Disorders, R=Revised, APA=American Psychiatric Association, AMA=American Medical Association, crit.=criteria
Questionnaires: AOD-S=Altomare's obstructed defecation score, BDQ=Bowel Disease Questionnaire, BSQ=Bowel Symptom Questionnaire, CSI=Children's Somatization Inventory, EGI=Questionnaire on esophago-gastrointestinal symptoms, FISI=Faecal Incontinence Severity Index, G-Q=Gastro-Questionnaire, GCSI=Gastroparesis Cardinal Symptom Index, GIM=Gastrointestinal Impairment Score, GIS=Gastrointestinal Symptom Score, GISS=GI Symptom Survey, IBS-Q=Irritable Bowe Syndrome Questionnaire, LDQ=Leeds Dyspepsia Questionnaire, MMPI=Minnesota Multiphasic Personality Inventory, Q-EWP=Questionnaire on Eating and Weight Patterns, ROME=Rome Diagnostic Questionnaire for FGIDs, SCL-90=Symptom Check List-90, SCQ=Self-constructed questionnaire, WCS=Wexner Constipation Score
AN=Anorexia nervosa (b/p=binge/purge subtype, r=restrictive subtype), ARFID=Avoidant/Restrictive Food Intake Disorder, BED=Binge-eating Disorder, BMI=body mass index, BN=Bulimia nervosa, BW=body weight, CT=computed tomography, FGID=Functional Gastrointestinal Disorder, GE=gastric emtying time, GI=gastrointestinal, IBS=Irritable Bowel Syndrome, IBW=ideal body weight, LES=lower esophagal sphincter, LF=Liquid formula, m=males, M=Medication, med.=median, n=sample size, MRI=magnetic resonance imaging, NIT=Non-invasive treatment, NA=Not applicable, NR=Not reported, NRP=Nutritional Rehabilitation program, O=objective, OSFED=Other SMA=Superior mesenteric artery syndrome, Specified Feeding and Eating Disorder, SSRI=Selective serotonine reuptake inhibitors, PT=Psychotherapy, S=subjective, UES=upper esophagal sphincter, UFED=Unspecified Feeding and Eating Disorder

Reference list

1. Abell TL, Malagelada JR, Lucas AR, Brown ML, Camilleri M, Go VL, Azpiroz F, Callaway CW, Kao PC, Zinsmeister AR, et al. Gastric electromechanical and neurohormonal function in anorexia nervosa. *Gastroenterology* (1987) 93:958–965.

2. Arii I, Yamashita T, Kinoshita M, Shimizu H, Nakamura M, Nakajima T. Treatment for inpatients with anorexia nervosa: Comparison of liquid formula with regular meals for improvement from emaciation. *Psychiatry Clin Neurosci* (1996) 50:55–59. doi:10.1111/j.1440-1819.1996.tb01664.x

3. Benini L, Todesco T, Grave RD, Deiorio F, Salandini L, Vantini I. Gastric emptying in patients with restricting and binge/purging subtypes of anorexia nervosa. *Am J Gastroenterol* (2004) 99:1448–1454. doi:10.1111/j.1572-0241.2004.30246.x

4. Benini L, Todesco T, Frulloni L, Dalle Grave R, Campagnola P, Agugiaro F, Cusumano CD, Gabbrielli A, Vantini I. Esophageal motility and symptoms in restricting and binge-eating/purging anorexia. *Dig Liver Dis* (2010) 42:767–772. doi:10.1016/j.dld.2010.03.018

5. Cuntz U, Enck P, Fruhauf E, Lehnert P, Riepl RL, Fichter MM, Otto B. Cholecystokinin Revisited: CCK and the Hunger Trap in Anorexia Nervosa. *PLoS One* (2013) 8: doi:10.1371/journal.pone.0054457

6. Heruc GA, Little TJ, Kohn M, Madden S, Clarke S, Horowitz M, Feinle-Bisset C. Appetite Perceptions, Gastrointestinal Symptoms, Ghrelin, Peptide YY and State Anxiety Are Disturbed in Adolescent Females with Anorexia Nervosa and Only Partially Restored with Short-Term Refeeding. *Nutrients* (2018) **11**: doi:10.3390/nu11010059

7. Holmes SR, Sabel AL, Gaudiani JL, Gudridge T, Brinton JT, Mehler PS. Prevalence and management of oropharyngeal dysphagia in patients with severe anorexia nervosa: A large retrospective review. *Int J Eat Disord* (2016) 49:159–166. doi:10.1002/eat.22441

8. Hotta M, Ohwada R, Akamizu T, Shibasaki T, Takano K, Kangawa K. Ghrelin Increases Hunger and Food Intake in Patients with Restricting-type Anorexia Nervosa: A Pilot Study. *Endocr J* (2009) 56:1119–1128. doi:10.1507/endocrj.K09E-168

9. Lee S, Ng KL, Kwok KPS, Thomas JJ, Becker AE. Gastrointestinal dysfunction in Chinese patients with fat-phobic and nonfat-phobic anorexia nervosa. *Transcult Psychiatry* (2012) 49:678–695. doi:10.1177/1363461512459487

10. Mack I, Cuntz U, Gramer C, Niedermaier S, Pohl C, Schwiertz A, Zimmermann K, Zipfel S, Enck P, Penders J. Weight gain in anorexia nervosa does not ameliorate the faecal microbiota, branched chain fatty acid profiles, and gastrointestinal complaints. *Sci Rep* (2016) 6: doi:10.1038/srep26752

11. Mattheus HK, Wagner C, Becker K, Buhren K, Correll CU, Egberts KM, Ehrlich S, Fleischhaker C, Focker M, Hahn F, et al. Incontinence and constipation in adolescent patients with anorexia nervosa-Results of a multicenter study from a German web-based registry for children and adolescents with anorexia nervosa. *Int J Eat Disord* (2019) doi:10.1002/eat.23182

12. Nakai Y, Nin K, Noma S, Hamagaki S, Takagi R, Teramukai S, Wonderlich SA. Clinical presentation and outcome of avoidant/restrictive food intake disorder in a Japanese sample. *Eat Behav* (2017) 24:49–53. doi:10.1016/j.eatbeh.2016.12.004

13. Ogawa A, Mizuta I, Fukunaga T, Takeuchi N, Honaga E, Sugita Y, Mikami A, Inoue Y, Takeda M. Electrogastrography abnormality in eating disorders. *Psychiatry Clin Neurosci* (2004) 58:300–310. doi:10.1111/j.1440-1819.2004.01236.x

14. Palla B, Litt IF. Medical complications of eating disorders in adolescents. *Pediatrics* (1988) 81:613–623.

15. Roberts MW, Li SH. Oral findings in anorexia nervosa and bulimia nervosa: a study of 47 cases. *J Am Dent Assoc* (1987) 115:407–410.

16. Santos CM, Cassiani RA, Dantas RO. VIDEOFLUOROSCOPIC EVALUATION OF SWALLOWS IN ANOREXIA NERVOSA. *Arq Gastroenterol* (2016) 53:136–140. doi:10.1590/s0004-28032016000300003

17. Scheutzel P, Gerlach U. Alpha-amylase isoenzymes in serum and saliva of patients with anorexia and bulimia nervosa. *Z Gastroenterol* (1991) 29:339–345.

18. Sileri P, Franceschilli L, De Lorenzo A, Mezzani B, Todisco P, Giorgi F, Gaspari AL, Jacoangeli F. Defecatory disorders in anorexia nervosa: a clinical study. *Tech Coloproctol* (2014) 18:439–444. doi:10.1007/s10151-013-1068-x

19. Szmukler GI, Young GP, Lichtenstein M, Andrews JT. A serial study of gastric emptying in anorexia nervosa and bulimia. *Intern Med J* (1990) 20:220–225.

20. Waldholtz BD, Andersen AE. Gastrointestinal symptoms in anorexia nervosa. A prospective study. *Gastroenterology* (1990) 98:1415–1419. doi:10.1016/0016-5085(90)91070-M

21. Winstead NS, Willard SG. Gastrointestinal complaints in patients with eating disorders. *J Clin Gastroenterol* (2006) 40:678–682. doi:10.1097/00004836-200609000-00003

22. Price C, Schmidt MA, Adam EJ, Lacey H. Parotid gland enlargement in eating disorders: an insensitive sign? *Eat Weight Disord* (2008) 13:e79-83.

23. Wolff HP, Henne G, Kruck F, Roscher S, Vecsei P, Brown JJ, Dusterdieck G, Lever AF, Robertson JI. Psychosomatic syndrome with gastrointestinal and-or renal potassium and sodium depletion, hyperreninnemia and secondary aldosteronism. *Schweiz Med Wochenschr* (1968) 98:1883–1892.

24. Abraham S, Luscombe GM, Kellow JE. Pelvic floor dysfunction predicts abdominal bloating and distension in eating disorder patients. *Scand J Gastroenterol* (2012) 47:625–631. doi:10.3109/00365521.2012.661762

25. Bluemel S, Menne D, Milos G, Goetze O, Fried M, Schwizer W, Fox M, Steingoetter A. Relationship of body weight with gastrointestinal motor and sensory function: studies in anorexia nervosa and obesity. *BMC Gastroenterol* (2017) 17:4. doi:10.1186/s12876-016-0560-y

26. Bozzato A, Burger P, Zenk J, Uter W, Iro H. Salivary gland biometry in female patients with eating disorders. *Eur Arch Otorhinolaryngol* (2008) 265:1095–1102. doi:10.1007/s00405-008-0598-8

27. Chiarioni G, Bassotti G, Monsignori A, Menegotti M, Salandini L, Di Matteo G, Vantini I, Whitehead WE. Anorectal dysfunction in constipated women with anorexia nervosa. *Mayo Clin Proc* (2000) 75:1015–1019. doi:10.4065/75.10.1015

28. Chun AB, Sokol MS, Kaye WH, Hutson WR, Wald A. Colonic and anorectal function in constipated patients with anorexia nervosa. *Am J Gastroenterol* (1997) 92:1879–1883.

29. Coddington RD, Bruch H. Gastric perceptivity in normal, obese and schizophrenic subjects. *Psychosomatics* (1970) 11:571–579.

30. Diamanti A, Bracci F, Gambarara M, Ciofetta GC, Sabbi T, Ponticelli A, Montecchi F, Marinucci S, Bianco G, Castro M. Gastric electric activity assessed by electrogastrography and gastric emptying scintigraphy in adolescents with eating disorders. *J Pediatr Gastroenterol Nutr* (2003) 37:35–41. doi:10.1097/00005176-200307000-00006

31. Domstad PA, Shih WJ, Humphries L, DeLand FH, Digenis GA. Radionuclide gastric emptying studies in patients with anorexia nervosa. *J Nucl Med* (1987) 28:816–819.

32. Dooley-Hash S, Lipson SK, Walton MA, Cunningham RM. Increased emergency department use by adolescents and young adults with eating disorders. *Int J Eat Disord* (2013) 46:308–315. doi:10.1002/eat.22070

33. Dubois A, Gross HA, Ebert MH, Castell DO. Altered gastric emptying and secretion in primary anorexia nervosa. *Gastroenterology* (1979) 77:319–323.

34. Fernandez-Aranda F, Aguera Z, Fernandez-Garcia J, Garrido-Sanchez L, Alcaide-Torres J, Tinahones F, Giner-Bartolome C, Banos RM, Botella C, Cebolla A, et al. Smell-taste dysfunctions in extreme weight/eating conditions: analysis of hormonal and psychological interactions. *Endocrine* (2016) 51:256–267. doi:10.1007/s12020-015-0684-9

35. Fisher MM, Rosen DS, Ornstein RM, Mammel KA, Katzman DK, Rome ES, Callahan ST, Malizio J, Kearney S, Walsh BT. Characteristics of Avoidant/Restrictive Food Intake Disorder in Children and Adolescents: A "New Disorder" in DSM-5. *J Adolesc Heal* (2014) 55:49–52. doi:10.1016/j.jadohealth.2013.11.013

36. Garcia Aroca J, Alonso Calderon JL, Garcia Redondo C, Rollan Villamarin V. Anorexia nervosa or somatic disease. *Cir Pediatr* (2001) 14:98–102.

37. Hill KK, Hill DB, Humphries LL, Maloney MJ, McClain CJ. A role forHelicobacter pylori in the gastrointestinal complaints of eating disorder patients? *Int J Eat Disord* (1999) 25:109–112. doi:10.1002/(SICI)1098-108X(199901)25:1<109::AID-EAT14>3.0.CO;2-D

38. Hirakawa M, Okada T, Iida M, Tamai H, Kobayashi N, Nakagawa T, Fujishima M. Small bowel transit time measured by hydrogen breath test in patients with anorexia nervosa. *Dig Dis Sci* (1990) 35:733–736.

39. Hutson WR, Wald A. Gastric emptying in patients with bulimia nervosa and anorexia nervosa. *Am J Gastroenterol* (1990) 85:41–46.

40. Kamal N, Chami T, Andersen A, Rosell FA, Schuster MM, Whitehead WE. Delayed gastrointestinal transit times in anorexia nervosa and bulimia nervosa. *Gastroenterology* (1991) 101:1320–1324.

41. Kinzl J, Biebl W, Herold M. Significance of vomiting for hyperamylasemia and sialadenosis in patients with eating disorders. *Int J Eat Disord* (1993) 13:117–124.

42. McCallum RW, Grill BB, Lange R, Planky M, Glass EE, Greenfeld DG. Definition of a gastric emptying abnormality in patients with anorexia nervosa. *Dig Dis Sci* (1985) 30:713–722.

43. Perez ME, Coley B, Crandall W, Di Lorenzo C, Bravender T. Effect of Nutritional Rehabilitation on Gastric Motility and Somatization in Adolescents with Anorexia. *J Pediatr* (2013) 163:867-+. doi:10.1016/j.jpeds.2013.03.011

44. Rigaud D, Bedig G, Merrouche M, Vulpillat M, Bonfils S, Apfelbaum M. Delayed gastric emptying in anorexia nervosa is improved by completion of a renutrition program. *Dig Dis Sci* (1988) 33:919–925.

45. Robinson PH. Gastric function in eating disorders. *Ann N Y Acad Sci* (1989) 575:455–456.

46. Saleh JW, Lebwohl P. Metoclopramide-induced gastric emptying in patients with anorexia nervosa. *Am J Gastroenterol* (1980) 74:127–132.

47. Salvioli B, Pellicciari A, Iero L, Di Pietro E, Moscano F, Gualandi S, Stanghellini V, De Giorgio R, Ruggeri E, Franzoni E. Audit of digestive complaints and psychopathological traits in patients with eating disorders: A prospective study. *Dig Liver Dis* (2013) 45:639–644. doi:10.1016/j.dld.2013.02.022

48. Satoh M, Yoshihara T. Clinical and ultracytochemical investigation of sialadenosis. *Acta Otolaryngol Suppl* (2004)122–127. doi:10.1080/03655230410017814

49. Sherman P, Leslie K, Golderg E, MacMillan J, Hunt R, Ernst P. Helicobacter pylori infection in adolescents with eating disorders and dyspeptic symptoms. *J Pediatr* (1993) 122:824–826.

50. Silverstone JT, Russell GF. Gastric "hunger" contractions in anorexia nervosa. *Br J Psychiatry* (1967) 113:257–263. doi:10.1192/bjp.113.496.257

51. Stacher G, Kiss A, Wiesnagrotzki S, Bergmann H, Hobart J, Schneider C. Oesophageal and gastric motility disorders in patients categorised as having primary anorexia nervosa. *Gut* (1986) 27:1120–1126.

52. Wockel L, Hummel T, Zepf FD, Jacob A, Poustka F. Changed taste perception in patients with eating disorders. *Z Kinder Jugendpsychiatr Psychother* (2007) 35:423–434. doi:10.1024/1422-4917.35.6.423

53. Chami TN, Andersen AE, Crowell MD, Schuster MM, Whitehead WE. GASTROINTESTINAL SYMPTOMS IN BULIMIA-NERVOSA - EFFECTS OF TREATMENT. *Am J Gastroenterol* (1995) 90:88–92.

54. DeJong H, Perkins S, Grover M, Schmidt U. The Prevalence of Irritable Bowel Syndrome in Outpatients with Bulimia Nervosa. *Int J Eat Disord* (2011) 44:661–664. doi:10.1002/eat.20901

55. Devlin MJ, Walsh BT, Guss JL, Kissileff HR, Liddle RA, Petkova E. Postprandial cholecystokinin release and gastric emptying in patients with bulimia nervosa. *Am J Clin Nutr* (1997) 65:114–120.

56. Devlin MJ, Kissileff HR, Zimmerli EJ, Samuels F, Chen BE, Brown AJ, Geliebter A, Walsh BT. Gastric emptying and symptoms of bulimia nervosa: Effect of a prokinetic agent. *Physiol Behav* (2012) 106:238–242. doi:10.1016/j.physbeh.2012.02.009

57. Geliebter A, Melton PM, McCray RS, Gallagher DR, Gage D, Hashim SA. Gastric capacity, gastric emptying, and test-meal intake in normal and bulimic women. *Am J Clin Nutr* (1992) 56:656–661.

58. Keel PK, Wolfe BE, Liddle RA, De Young KP, Jimerson DC. Clinical features and physiological response to a test meal in purging disorder and bulimia nervosa. *Arch Gen Psychiatry* (2007) 64:1058–1066. doi:10.1001/archpsyc.64.9.1058

59. Kiss A, Wiesnagrotzki S, Abatzi TA, Meryn S, Haubenstock A, Base W. Upper gastrointestinal endoscopy findings in patients with long-standing bulimia nervosa. *Gastrointest Endosc* (1989) 35:516–518.

60. Koch KL, Bingaman S, Tan L, Stern RM. Visceral perceptions and gastric myoelectrical activity in healthy women and in patients with bulimia nervosa. *Neurogastroenterol Motil* (1998) 10:3–10. doi:10.1046/j.1365-2982.1998.00080.x

61. Metzger ED, Levine JM, McArdle CR, Wolfe BE, Jimerson DC. Salivary gland enlargement and elevated serum amylase in bulimia nervosa. *Biol Psychiatry* (1999) 45:1520–1522.

62. Mond JM, Myers TC, Crosby RD, Hay PJ, Mitchell JE. Bulimic Eating Disorders in Primary Care: Hidden Morbidity Still? *J Clin Psychol Med Settings* (2010) 17:56–63. doi:10.1007/s10880-009-9180-9

63. Nickl NJ, Brazer SR, Rockwell K, Smith JW. Patterns of esophageal motility in patients with stable bulimia. *Am J Gastroenterol* (1996) 91:2544–2547.

64. Ogren FP, Huerter J V, Pearson PH, Antonson CW, Moore GF. Transient salivary gland hypertrophy in bulimics. *Laryngoscope* (1987) 97:951–953.

65. Paszynska E, Jurga-Krokowicz J, Shaw H. The use of parotid gland activity analysis in patients with gastro-esophageal reflux disease (GERD) and bulimia nervosa. *Adv Med Sci* (2006) 51:208–213.

66. Paszynska E, Linden RW, Slopien A, Rajewski A. Parotid gland flow activity and inorganic composition in purging bulimic patients treated with fluoxetine. *World J Biol Psychiatry* (2013) 14:634–639. doi:10.3109/15622975.2013.795242

67. Riad M, Barton JR, Wilson JA, Freeman CP, Maran AG. Parotid salivary secretory pattern in bulimia nervosa. *Acta Otolaryngol* (1991) 111:392–395.

68. Roberts MW, Tylenda CA, Sonies BC, Elin RJ. Dysphagia in bulimia nervosa. *Dysphagia* (1989) 4:106–111.

69. Robertson C, Millar H. Hyperamylasemia in bulimia nervosa and hyperemesis gravidarum. *Int J Eat Disord* (1999) 26:223–227.

70. Rothstein SG. Reflux and vocal disorders in singers with bulimia. *J Voice* (1998) 12:89–90.

71. Rytomaa I, Jarvinen V, Kanerva R, Heinonen OP. Bulimia and tooth erosion. *Acta Odontol Scand* (1998) 56:36–40.

72. Shih WJ, Humphries L, Digenis GA, Castellanos FX, Domstad PA, DeLand FH. Tc-99m labeled triethelene tetraamine polysterene resin gastric emptying studies in bulimia patients. *Eur J Nucl Med* (1987) 13:192–196.

73. Tylenda CA, Roberts MW, Elin RJ, Li SH, Altemus M. Bulimia nervosa. Its effect on salivary chemistry. *J Am Dent Assoc* (1991) 122:37–41.

74. Valena V, Young WG. Dental erosion patterns from intrinsic acid regurgitation and vomiting. *Aust Dent J* (2002) 47:106–115.

75. Walsh BT, Wong LM, Pesce MA, Hadigan CM, Bodourian SH. Hyperamylasemia in bulimia nervosa. *J Clin Psychiatry* (1990) 51:373–377.

76. Walsh BT, Zimmerli E, Devlin MJ, Guss J, Kissileff HR. A disturbance of gastric function in bulimia nervosa. *Biol Psychiatry* (2003) 54:929–933. doi:10.1016/s0006-3223(03)00176-8

77. Zimmerli EJ, Walsh BT, Guss JL, Devlin MJ, Kissileff HR. Gastric compliance in bulimia nervosa. *Physiol Behav* (2006) 87:441–446. doi:10.1016/j.physbeh.2005.11.010

78. Cremonini F, Camilleri M, Clark MM, Beebe TJ, Locke GR, Zinsmeister AR, Herrick LM, Talley NJ. Associations among binge eating behavior patterns and gastrointestinal symptoms: a population-based study. *Int J Obes* (2009) 33:342–353. doi:10.1038/ijo.2008.272

79. Crowell MD, Cheskin LJ, Musial F. PREVALENCE OF GASTROINTESTINAL SYMPTOMS IN OBESE AND NORMAL-WEIGHT BINGE EATERS. *Am J Gastroenterol* (1994) 89:387–391.

80. Geliebter A, Yahav EK, Gluck ME, Hashim SA. Gastric capacity, test meal intake, and appetitive hormones in binge eating disorder. *Physiol Behav* (2004) 81:735–740. doi:10.1016/j.physbeh.2004.04.014

81. Gowen GF, Stoldt HS, Rosato FE. Five risk factors identify patients with gastroesophageal intussusception. *Arch Surg* (1999) 134:1394–1397.

82. Levy RL, Linde JA, Feld KA, Crowell MD, Jeffery RW. The association of gastrointestinal symptoms with weight, diet, and exercise in weight-loss program participants. *Clin Gastroenterol Hepatol* (2005) 3:992–996. doi:10.1053/s1542-3565(05)00696-8

83. Peat CM, Huang L, Thornton LM, Von Holle AF, Trace SE, Lichtenstein P, Pedersen NL, Overby DW, Bulik CM. Binge eating, body mass index, and gastrointestinal symptoms. *J Psychosom Res* (2013) 75:456–461. doi:10.1016/j.jpsychores.2013.08.009

84. Thornton LM, Watson HJ, Jangmo A, Welch E, Wiklund C, von Hausswolff-Juhlin Y, Norring C, Herman BK, Larsson H, Bulik CM. Binge-eating disorder in the Swedish national registers: Somatic comorbidity. *Int J Eat Disord* (2017) 50:58–65. doi:10.1002/eat.22624

85. Thornton LM, Trace SE, Brownley KA, Ålgars M, Mazzeo SE, Bergin JE, Maxwell M, Lichtenstein P, Pedersen NL, Bulik CM. A Comparison of Personality, Life Events, Comorbidity, and Health in Monozygotic Twins Discordant for Anorexia Nervosa. *Twin Res Hum Genet* (2017) **20**:310–318. doi:10.1017/thg.2017.27

86. Lobera IJ, Santed MA, Rios PB. Impact of functional dyspepsia on quality of life in eating disorder patients: the role of thought-shape fusion. *Nutr Hosp* (2011) 26:1363–1371. doi:10.3305/nh.2011.26.6.5288

87. Brook GK. Acute gastric dilatation in anorexia nervosa. *Br Med J* (1977) 2:499–500.

88. Buchman AL, Ament ME, Weiner M, Kodner A, Mayer EA. Reversal of megaduodenum and duodenal dysmotility associated with improvement in nutritional status in primary anorexia nervosa. *Dig Dis Sci* (1994) 39:433–440.

89. El Ghoch M, Benini L, Sgarbi D, Dalle Grave R. Solitary rectal ulcer syndrome in a patient with anorexia nervosa: A case report. *Int J Eat Disord* (2016) 49:731–735. doi:10.1002/eat.22548

90. Elizondo-Romo R, Nunez-Zuno JA, Jimenez-Gonzalez IJ, Montiel-Nunez E, Jimenez JV, Carrillo-Perez DL. Air in the portal space: where does it come from? *Postgrad Med J* (2018) **94**:665. doi:10.1136/postgradmedj-2018-135845

91. Evans DS. Acute dilatation and spontaneous rupture of the stomach. *Br J Surg* (1968) 55:940–942.

92. Fenley J, Powers PS, Miller J, Rowland M. Untreated anorexia nervosa. A case study of the medical consequences. *Gen Hosp Psychiatry* (1990) 12:264–270.

93. Fernandez Lopez MT, Lopez Otero MJ, Bardasco Alonso ML, Alvarez Vazquez P, Rivero Luis MT, Garcia Barros G. Wilkie syndrome: report of a case. *Nutr Hosp* (2011) 26:646–649. doi:10.1590/s0212-16112011000300031

94. Garcia X, Soler L, Amer M, Camacho J, Arnalich F. Twenty one year old male with cachexia and presence of abundant amount of intraabdominal gas. *Rev Clin Esp* (2006) 206:355–356.

95. Goldstein MA, Herzog DB, Misra M, Sagar P. Case records of the Massachusetts General Hospital. Case 29-2008. A 19-year-old man with weight loss and abdominal pain. *N Engl J Med* (2008) 359:1272–1283. doi:10.1056/NEJMcpc0804641

96. Gwee K, Teh A, Huang C. Acute superior mesenteric artery syndrome and pancreatitis in anorexia nervosa. *Australas Psychiatry* (2010) 18:523–526. doi:10.3109/10398562.2010.498885

97. Holmes SR, Gudridge TA, Gaudiani JL, Mehler PS. Dysphagia in severe anorexia nervosa and potential therapeutic intervention: a case series. *Ann Otol Rhinol Laryngol* (2012) 121:449–456. doi:10.1177/000348941212100705

98. Masuda A, Nakano T, Uehara H, Kuroki K, Tei C. Hyperbaric oxygen for anorexia nervosa. *Intern Med* (2001) 40:635–637.

99. Osegueda de Rodriguez EJ, Hernandez-Villegas AC, Serralde-Zuniga AE, Reyes-Ramirez ALDC. The two sides of superior mesenteric artery syndrome treatment: conservative or surgical management? *Nutr Hosp* (2017) **34**:997–1000. doi:10.20960/nh.1006

100. Saito S, Kita K, Morioka CY, Watanabe A. Rapid recovery from anorexia nervosa after a life-threatening episode with severe thrombocytopenia: report of three cases. *Int J Eat Disord* (1999) 25:113–118.

101. Saito S, Haruta H, Kobayashi T, Kato S. A Case of Anorexia Nervosa With Tracheoesophageal Fistula. *Psychosomatics* (2015) 56:419–422. doi:10.1016/j.psym.2014.05.023

102. Sansone RA, Sansone LA. Metoclopramide and unintended weight gain. *Int J Eat Disord* (2003) 34:265–268. doi:10.1002/eat.10181

103. Abdu RA, Garritano D, Culver O. Acute gastric necrosis in anorexia nervosa and bulimia. Two case reports. *Arch Surg* (1987) 122:830–832.

104. Adson DE, Mitchell JE, Trenkner SW. The superior mesenteric artery syndrome and acute gastric dilatation in eating disorders: a report of two cases and a review of the literature. *Int J Eat Disord* (1997) 21:103–114.

105. Albano MN, Costa Almeida C, Louro JM, Martinez G. Increase body weight to treat superior mesenteric artery syndrome. *BMJ Case Rep* (2017) 2017: doi:10.1136/bcr-2017-219378

106. Berke GS, Calcaterra TC. Parotid hypertrophy with bulimia: a report of surgical management. *Laryngoscope* (1985) 95:597–598.

107. Birmingham CL, Cardew S, Gritzner S. Gastric bezoar in anorexia nervosa. *Eat Weight Disord* (2007) 12:e28-9.

108. Browning CH. Anorexia nervosa: complications of somatic therapy. *Compr Psychiatry* (1977) 18:399–403.

109. Bruno V, Amato M, Catapano S, Iovino P. Dental erosion in patients seeking treatment for gastrointestinal complaints: a case series. *J Med Case Rep* (2015) 9:250. doi:10.1186/s13256-015-0738-x

110. Buchanan JA, Fortune F. Bilateral parotid enlargement as a presenting feature of bulimia nervosa in a post-adolescent male. *Postgr Med J* (1994) 70:27–30.

111. De Caprio C, Pasanisi F, Contaldo F. Gastrointestinal complications in a patient with eating disorders. *Eat Weight Disord* (2000) 5:228–230.

112. di Luca A, Ricci E, Grassi VM, Arena V, Oliva A. An Exceptional Case of Acute Respiratory Failure Caused by Intra-Thoracic Gastric Perforation Secondary to Overeating. *J Forensic Sci* (2019) **64**:292–294. doi:10.1111/1556-4029.13825

113. Dumouchel J, Lvovschi V, Joly LM. Obstructive acute renal failure by severe gastric distension after binge. *Am J Emerg Med* (2017) 35:1210.e5-1210.e7. doi:10.1016/j.ajem.2017.03.077

114. Eraslan D, Ozturk O, Bor S. Eating Disorder Symptoms Improved by Antireflux Surgery: A Case Report with a Six-Year Follow Up. *Isr J Psychiatry Relat Sci* (2009) 46:231–235.

115. Franco-Lopez A, Badillo S, Contreras J. Acute gastric dilatation in a bulimic patient; systemic efects. *Nutr Hosp* (2012) 27:1364–1367. doi:10.3305/nh.2012.27.4.5873

116. Gore RM, Port RB. Progressive antral narrowing in an adolescent. *Jama* (1982) 247:73–74.

117. Hollatz F, Ziolko HU. The differential diagnosis of anorexia nervosa. Coincidence of somatic disease and psychogenic emaciation (author's transl). *MMW Munch Med Wochenschr* (1976) 118:263–266.

118. Imai T, Michizawa M. Necrotizing sialometaplasia in a patient with an eating disorder: palatal ulcer accompanied by dental erosion due to binge-purging. *J Oral Maxillofac Surg* (2013) 71:879–885. doi:10.1016/j.joms.2012.10.033

119. Kim SC, Cho HJ, Kim MC, Ko YG. Sudden cardiac arrest due to acute gastric dilatation in a patient with an eating disorder. *Emerg Med J* (2009) 26:227–228. doi:10.1136/emj.2008.065391

120. Kim HH, Park SJ, Park MI, Moon W. Acute gastric dilatation and acute pancreatitis in a patient with an eating disorder: solving a chicken and egg situation. *Intern Med* (2011) 50:571–575.

121. Koyazounda A, Le Baron JC, Abed N, Daussy D, Lafarie M, Pinsard M. Gastric necrosis caused by acute gastric dilatation. Total gastrectomy. Recovery. *J Chir* (1985) 122:403–407.

122. Lin CY, Lee CS, Lin DY, Hong CF, Jan YY, Lin PY, Chen PC, Wu CS. Emphysematous gastritis secondary to acute gastric dilatation. *J Gastroenterol Hepatol* (1995) 10:612–615.

123. Mascolo M, Dee E, Townsend R, Brinton JT, Mehler PS. Severe gastric dilatation due to superior mesenteric artery syndrome in anorexia nervosa. *Int J Eat Disord* (2015) 48:532–534. doi:10.1002/eat.22385

124. Mathevon T, Rougier C, Ducher E, Pic D, Garcier JM, Schmidt J. Acute abdominal dilatation, a serious complication in the case of anorexia nervosa. *Press Med* (2004) 33:601–603.

125. Matsuyama T, Komeda S, Nobayashi M, Imanishi M, Kawaguchi S. Acute gastric dilatation causing bacterial cerebral aneurysm--case report. *Int J Eat Disord* (2008) 41:380–382. doi:10.1002/eat.20487

126. Mehler PS, Weiner KL. Use of total parenteral nutrition in the refeeding of selected patients with severe anorexia nervosa. *Int J Eat Disord* (2007) 40:285–287. doi:10.1002/eat.20371

127. Mignogna MD, Fedele S, Lo Russo L. Anorexia/bulimia-related sialadenosis of palatal minor salivary glands. *J Oral Pathol Med* (2004) 33:441–442. doi:10.1111/j.1600-0714.2004.00208.x

128. Mitchell N, Norris ML. Rectal prolapse associated with anorexia nervosa: a case report and review of the literature. *J Eat Disord* (2013) 1:39. doi:10.1186/2050-2974-1-39

129. Pacciardi B, Cargioli C, Mauri M. Barrett's esophagus in anorexia nervosa: a case report. *Int J Eat Disord* (2015) 48:147–150. doi:10.1002/eat.22288

130. Pandey R, Maqbool A, Jayachandran N. Medical image. Massive gastric dilatation secondary to a binge episode in bulimia nervosa. *N Z Med J* (2009) 122:85–86.

131. Repesse X, Bodson L, Au SM, Charron C, Vieillard-Baron A. Gastric dilatation and circulatory collapse due to eating disorder. *Am J Emerg Med* (2013) 31:633.e3–4. doi:10.1016/j.ajem.2012.10.018

132. Roggo A, Filippini L. Transmural esophagus perforation. Experiences with conservative treatment. *Schweiz Med Wochenschr* (1989) 119:1257–1263.

133. Roseborough GS, Felix WA. Disseminated intravascular coagulation complicating gastric perforation in a bulimic woman. *Can J Surg* (1994) 37:55–58.

134. Sastre JA, López T, Garzón JC. Young adult with abdominal pain. *Ann Emerg Med* (2015) 66:e1–e2. doi:10.1016/j.annemergmed.2015.05.006

135. Schechter JO, Altemus M, Greenfeld DG. Food bingeing and esophageal perforation in anorexia nervosa. *Hosp Community Psychiatry* (1986) 37:507–508.

136. Sinicina I, Pankratz H, Buttner A, Mall G. Death due to neurogenic shock following gastric rupture in an anorexia nervosa patient. *Forensic Sci Int* (2005) 155:7–12. doi:10.1016/j.forsciint.2004.10.021

137. Trott GE, Elliger T, Kerscher P, Nissen G. Acute abdomen in anorexia nervosa. A case report. *Fortschr Med* (1990) 108:525–526.

138. Tweed-Kent AM, Fagenholz PJ, Alam HB. Acute gastric dilatation in a patient with anorexia nervosa binge/purge subtype. *J Emerg Trauma Shock* (2010) 3:403–405. doi:10.4103/0974-2700.70774

139. van Dijk JP, van den Akker L, Barwegen MG. Fatal outcome of spontaneous rupture of the stomach in a patient with anorexia nervosa. *Eur J Surg* (1994) 160:699–700.

140. van Eetvelde E, Verfaillie L, Van De Winkel N, Hubloue I. Acute gastric dilatation causing acute limb ischemia in an anorexia nervosa patient. *J Emerg Med* (2014) 46:e141-3. doi:10.1016/j.jemermed.2013.11.093

141. Walsh BT, Croft CB, Katz JL. Anorexia nervosa and salivary gland enlargement. *Int J Psychiatry Med* (1981) 11:255–261.

142. Watanabe S, Terazawa K, Asari M, Matsubara K, Shiono H, Shimizu K. An autopsy case of sudden death due to acute gastric dilatation without rupture. *Forensic Sci Int* (2008) 180:e6–e10. doi:10.1016/j.forsciint.2008.07.005

143. Willeke F, Riedl S, von Herbay A, Schmidt H, Hoffmann V, Stern J. Decompensated acute gastric dilatation caused by a bulimic attack in anorexia nervosa. *Dtsch Med Wochenschr* (1996) 121:1220–1225. doi:10.1055/s-2008-1043130

144. Yamada Y, Nishimura S, Inoue T, Tsujimura T, Fushimi H. Anorexia nervosa with ischemic necrosis of the segmental ileum and cecum. *Intern Med* (2001) 40:304–307.

145. Yamaguchi H, Arita Y, Hara Y, Kimura T, Nawata H. ANOREXIA-NERVOSA RESPONDING TO ZINC SUPPLEMENTATION - A CASE-REPORT. *Gastroenterol Jpn* (1992) 27:554–558.

146. de Silva AP, Molagoda A, Fernando PL, de Silva HJ. The young woman who could not stop vomiting. *Postgr Med J* (1998) 74:691–692.

147. Diamanti A, Basso MS, Cecchetti C, Monti L, Noto C, De Maria F, Castro M. Digestive complication in severe malnourished anorexia nervosa patient: a case report of necrotizing colitis. *Int J Eat Disord* (2011) 44:91–93. doi:10.1002/eat.20778

148. Dreznik Z, Vishne TH, Kristt D, Alper D, Ramadan E. Rectal prolapse: a possibly underrecognized complication of anorexia nervosa amenable to surgical correction. *Int J Psychiatry Med* (2001) 31:347–352. doi:10.2190/3987-2n5a-fjdg-m89f

149. Dzirlo L, Haunold I, Lung S, Buchinger E, Muller-Knespel EM, Weiss P. Pneumatosis intestinalis in anorexia nervosa: a case report. *Z Gastroenterol* (2013) 51:1265–1268. doi:10.1055/s-0031-1281680

150. Elbadawy MH. Chronic superior mesenteric artery syndrome in anorexia nervosa. *Br J Psychiatry* (1992) 160:552–554.

151. Kalouche I, Leturgie C, Tronc F, Bokobza B, Michot F, Pons P, Menard G. The superior mesenteric artery syndrome. Apropos of a case and review of the literature. *Ann Chir* (1991) 45:609–612.

152. Kaye JC, Madden M V, Leaper DJ. Anorexia nervosa and necrotizing colitis. *Postgr Med J* (1985) 61:41–42.

153. Mearelli F, Degrassi F, Occhipinti AA, Casarsa C, De Manzini N, Biolo G. Pinched: superior mesenteric artery syndrome. *Am J Med* (2014) 127:393–394. doi:10.1016/j.amjmed.2014.01.008

154. Miller TJ, Kuhlman JE, Fishman EK. Mesenteric volvulus in a patient with anorexia nervosa. *South Med J* (1991) 84:263–265.

155. Pua U. Multisegment jejunojejunal intussusception in gastrojejunostomy. *Med J Aust* (2011) 195:148.

156. Sakka S, Hurst P, Khawaja H. Anorexia nervosa and necrotizing colitis: case report and review of the literature. *Postgr Med J* (1994) 70:369–370.

157. Vannatta JB, Cagas CR, Cramer RI. Superior mesenteric artery (Wilkie's) syndrome: report of three cases and review of the literature. *South Med J* (1976) 69:1461–1465.

158. Wu D, Guan H. Hepatic portal venous gas in anorexia nervosa. *Qjm* (2016) 109:629–630. doi:10.1093/qjmed/hcw097

159. Yao SY, Mikami R, Mikami S. Minimally invasive surgery for superior mesenteric artery syndrome: A case report. *World J Gastroenterol* (2015) 21:12970–12975. doi:10.3748/wjg.v21.i45.12970

160. Antic M, Ghodduci KM, Brussaard C, de Mey J. Massive gastric dilatation in bulimic patient. *Jbr-btr* (2014) 97:136–137.

161. Beiles CB, Rogers G, Upjohn J, Wise AG. Gastric dilatation and necrosis in bulimia: a case report. *Australas Radiol* (1992) 36:75–76.

162. Carlson DL. Necrotizing sialometaplasia: a practical approach to the diagnosis. *Arch Pathol Lab Med* (2009) 133:692–698. doi:10.1043/1543-2165-133.5.692

163. Delap TG, Grant WE, Dick R, Quiney RE. Retropharyngeal abscess-an unusual complication of anorexia nervosa. *J Laryngol Otol* (1996) 110:483–484.

164. Devitt PG, Stamp GW. Acute clostridial enteritis--or pig-bel? *Gut* (1983) 24:678–679.

165. Maung H, Buxey KN, Studd C, Ket S. Acute gastric dilatation in a bulimic patient. *Gastrointest Endosc* (2017) 85:455–457. doi:10.1016/j.gie.2016.03.015

166. Navab F, Avunduk C, Gang D, Frankel K. Bulimia nervosa complicated by Barrett's esophagus and esophageal cancer. *Gastrointest Endosc* (1996) 44:492–494.

167. Patocskai EJ, Thomas JM. Gastric necrosis in a patient with bulimia. *Eur J Surg* (2002) 168:302–304. doi:10.1002/ejs.50

168. Petrin C, Tacchetti G, Preciso G, Gallo F, Bernardi S, Mion M. Acute distension followed by gastric rupture after an episode of bulimia. Apropos of a case. *J Chir* (1990) 127:213–215.

169. Reichel O, Mayr D, Wollenberg B. Recurring bilateral parotid swelling. *HNO* (2003) 51:1002–1004. doi:10.1007/s00106-003-0878-y

170. Rosset N, Yuen B. Acute massive gastric dilatation with abdominal compartment syndrome in a patient with bulimia nervosa. *Intensive Care Med* (2015) 41:2189–2190. doi:10.1007/s00134-015-3875-1

171. Bravender T, Story L. Massive binge eating, gastric dilatation and unsuccessful purging in a young woman with bulimia nervosa. *J Adolesc Heal* (2007) 41:516–518. doi:10.1016/j.jadohealth.2007.06.018

172. Cosins JM, Frederickx Y, Yousif A, Hamoir M, Van den Eeckhaut J. Mannequin syndrome. *Acta Otorhinolaryngol Belg* (1986) 40:678–681.

173. Cuellar RE, Kaye WH, Hsu LK, Van Thiel DH. Upper gastrointestinal tract dysfunction in bulimia. *Dig Dis Sci* (1988) 33:1549–1553.

174. Czarnecki CA, O'Coclain DF. Images in clinical medicine. Severe abdominal pain in a girl. *N Engl J Med* (2002) 347:e3. doi:10.1056/ENEJMicm020114

175. Elsharif M, Doulias T, Aljundi W, Balchandra S. Abdominal aortic occlusion and vascular compromise secondary to acute gastric dilatation in a patient with bulimia. *Ann R Coll Surg Engl* (2014) 96:e15-7. doi:10.1308/003588414x13946184902848

176. Garcia Vasquez C, Cortes Guiral D, Rivas Fidalgo S, Celdran Uriarte A. Gigantic gastric retention due to bulimic binge eating. *Cir Esp* (2014) 92:e33. doi:10.1016/j.ciresp.2012.10.017

177. Herrlinger P, Gundlach P. Hypertrophy of the salivary glands in bulimia. *HNO* (2001) 49:557–559.

178. Hohenauer P, Dunser MW. Massive gastric distension. *Wien Klin Wochenschr* (2011) 123:592. doi:10.1007/s00508-011-1599-y

179. Jones WR, Morgan JF. Eructophilia in bulimia nervosa: A clinical feature. *Int J Eat Disord* (2012) **45**:298–301. doi:10.1002/eat.20919

180. Kashyap AS, Chopra D, Anand KP, Arora S, Kashyap S. Acute gastric dilatation. *Emerg Med J* (2009) 26:326. doi:10.1136/emj.2008.062356

181. Kim SP, Kim SJ, Sun KH, Park Y. A Case of Gastric Atony on Bulimia Nervosa. *Iran Red Crescent Med J* (2017) 19: doi:10.5812/ircmj.13065

182. Kimura A, Masuda N, Haga N, Ito T, Otsuka K, Takita J, Satomura H, Kumakura Y, Kato H, Kuwano H. Gastrojejunostomy for pyloric stenosis after acute gastric dilatation due to overeating. *World J Gastroenterol* (2015) 21:1670–1674. doi:10.3748/wjg.v21.i5.1670

183. Lambeck W, Hacki T. Voice disorder and bulimia. *HNO* (1997) 45:36–39.

184. Levin PA, Falko JM, Dixon K, Gallup EM, Saunders W. Benign parotid enlargement in bulimia. *Ann Intern Med* (1980) 93:827–829.

185. Malik M, Stratton J, Sweeney WB. Rectal prolapse associated with bulimia nervosa: report of seven cases. *Dis Colon Rectum* (1997) 40:1382–1385.

186. Mitchell JE, Pyle RL, Miner RA. Gastric dilatation as a complication of bulimia. *Psychosomatics* (1982) 23:96–97. doi:10.1016/s0033-3182(82)70818-7

187. Park KK, Tung RC, de Luzuriaga AR. Painful parotid hypertrophy with bulimia: a report of medical management. *J Drugs Dermatol* (2009) 8:577–579.

188. Pedrolli C, Sacchi MC, Togni M, Cereda E. A Case of Hyperemesis in Bulimia Nervosa. *Int J Eat Disord* (2015) 48:446–448. doi:10.1002/eat.22315

189. Sansone RA, Naqvi A, Sansone LA. An unusual cause of dizziness in bulimia nervosa: A case report. *Int J Eat Disord* (2005) 37:364–366. doi:10.1002/eat.20095

190. Schoning H, Emshoff R, Kreczy A. Necrotizing sialometaplasia in two patients with bulimia and chronic vomiting. *Int J Oral Maxillofac Surg* (1998) 27:463–465.

191. Taylor VE, Sneddon J. Bilateral facial swelling in bulimia. *Br Dent J* (1987) 163:115–117.

192. Usui A, Kawasumi Y, Ishizuka Y, Hosokai Y, Ikeda T, Saito H, Funayama M. A Case Report of Postmortem Radiography of Acute, Fatal Abdominal Distension After Binge Eating. *Am J Forensic Med Pathol* (2016) 37:223–226. doi:10.1097/paf.0000000000000243

193. Vavrina J, Muller W, Gebbers JO. Enlargement of salivary glands in bulimia. *J Laryngol Otol* (1994) 108:516–518.

194. Wilson T, Price T. Revisiting a controversial surgical technique in the treatment of bulimic parotid hypertrophy. *Am J Otolaryngol* (2003) 24:85–88. doi:10.1053/ajot.2003.22

195. Winstead DK, Willard SG. Bulimia: diagnostic clues. *South Med J* (1983) 76:313–315.
